# Phosphorescence of Heavy, T‐Shaped Pnictogen Trisamides in Solution at Room Temperature in the Near‐Infrared II Region

**DOI:** 10.1002/chem.71049

**Published:** 2026-05-01

**Authors:** Katharina L. Deuter, Sotirios Pavlidis, Rainer F. Winter, Peter Coburger, Josh Abbenseth

**Affiliations:** ^1^ Faculty for Chemistry University of Konstanz Konstanz Germany; ^2^ Institut für Chemie Humboldt‐Universität zu Berlin Berlin Germany; ^3^ Department of Inorganic Chemistry TU München Garching Germany; ^4^ Department of Chemistry The University of Manchester Manchester United Kingdom

**Keywords:** NIR emitter, photoluminescence, pincer ligand, pnictogens, redox non‐innocence

## Abstract

We report T‐shaped Sb(III) and Bi(III) trisamides that exhibit phosphorescence in the near‐infrared‐II region (NIR‐II, 1000–1700 nm) in solution at 25 °C. Geometric constraint of the pnictogen(III) centers by an NNN pincer ligand enables ligand‐to‐metal charge transfer upon visible‐light excitation. Significant structural reorganization upon photoexcitation leads to the largest Stokes shifts (> 6000 cm^−1^) ever reported for Sb and Bi compounds to emit in the NIR region, reminiscent of precious metal photosensitizers. The emissive triplet states exhibit lifetimes up to the microsecond range, offering a new design principle for NIR emitters based on main group elements.

## Graphical abstract

The unique photophysical properties of heavy T‐shaped Sb and Bi trisamides are reported. A ligand‐to‐pnictogen charge‐transfer excitation from a redox‐active NNN pincer to a vacant pnictogen p‐orbital populates a triplet state that emits in the highly desirable NIR‐II region, with lifetimes up to the µs range. This represents the first example of Sb(III) and Bi(III) compounds showing NIR emission in solution at room temperature.

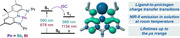

## 1 Introduction

Near infrared (NIR) triplet emitters are crucial for the future development of biomedical imaging, remote sensing, optical communication, and many more applications. To date, most NIR emitters are based either on large conjugated organic π‐systems or on complexes of rare noble transition metals such as platinum, osmium, or iridium [1, 2, 3, 4, 5, 6, 7, 8]. Organic NIR emitters often suffer from low efficacy due to π‐stacking in the solid state and strong dipole‐dipole interactions which have adverse effects on the lifetime of the emissive state as well as the overall quantum yields. Several classes of efficient organic NIR emitters are nevertheless known, however they usually emit by fluorescence [9, 10, 11, 12, 13, 14]. Their longer‐lived excited states render phosphorescent NIR emitters advantageous for advanced applications [7, 8], as can be found among transition metal complexes with push‐pull chromophores or large π‐conjugated ligands, particularly those of platinum [15, 16, 17, 18, 19, 20]. Phosphorescent compounds based on main group elements offer an attractive alternative in this regard but remain severely underexplored [21]. Antimony and bismuth are especially interesting in this context owing to their high affordability and comparably low toxicity [22, 23, 24, 25, 26]. In addition, the influence of relativistic effects on the photophysical properties of p‐block based NIR emitters can be studied upon comparing isostructural Sb and Bi compounds, providing crucial structure‐reactivity insights [22, 23, 24, 25, 26, 27].

**FIGURE 1:**
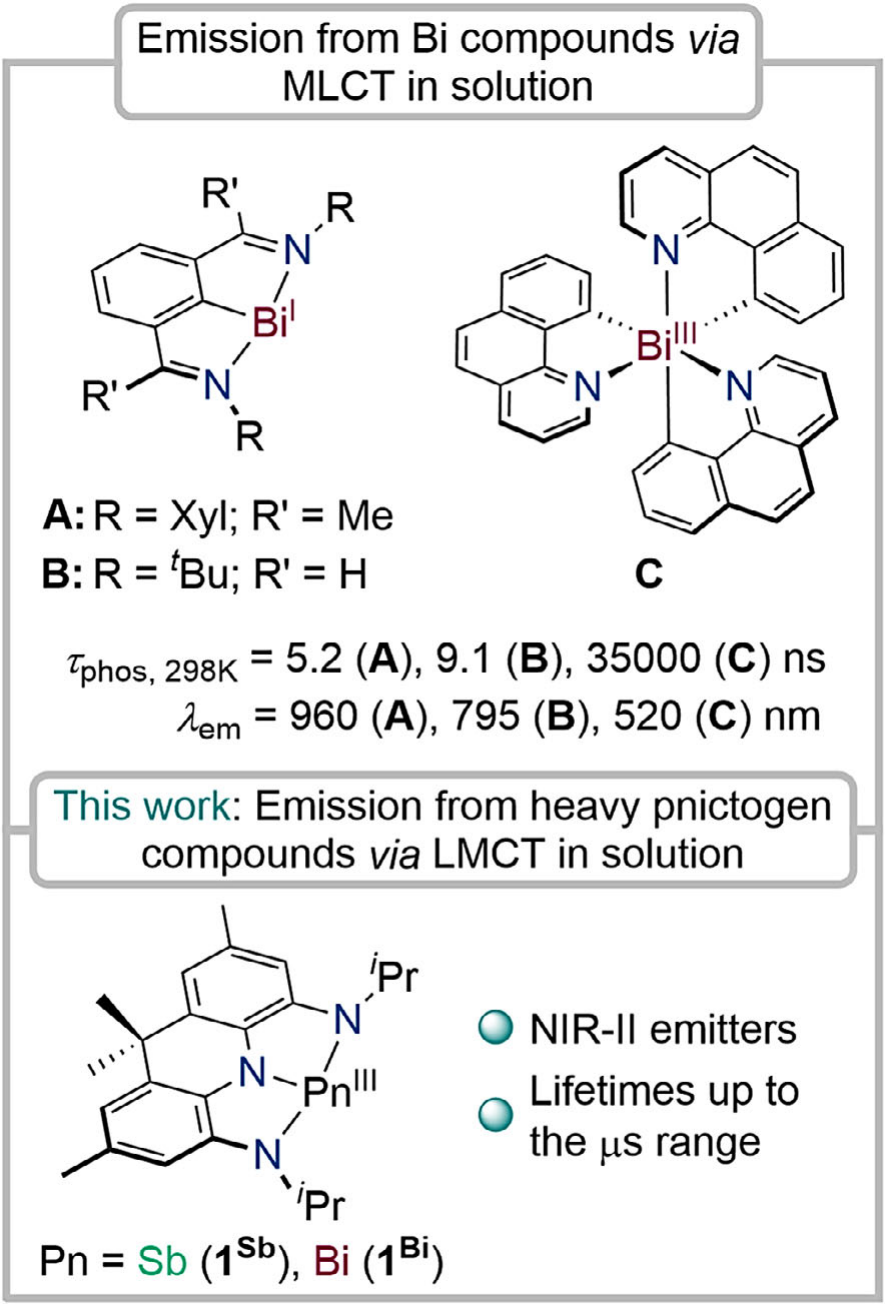
Recently reported Bi(I) NCN pincer compounds (**A**, **B**) and Bi(bzq)_3_ (**C**) and their photochemical properties (top); Key features of the Sb(III) and Bi(III) NNN pincer compounds **1^Sb^** and **1^Bi^** reported in this study (bottom).

The field of NIR emitters based on heavy pnictogens is still in its nascent stages, with only two examples restricted to Bi(I) species. The groups of Cornella and Winter reported on the photoluminescent properties of the bismuthinidenes **A** and **B** that are intensely colored in solution (Figure 1) [28, 29]. For both species, the highest occupied molecular orbital (HOMO) possesses large Bi(6p)‐orbital character, while the lowest unoccupied molecular orbital (LUMO) is ligand‐based. Excitation by visible light initiates a metal‐to‐ligand charge transfer (MLCT) process from the singlet ground state S_0_ to an excited singlet state S_1_. This is followed by rapid intersystem crossing (ISC) to yield an emissive triplet ^3^MLCT state T_1_ with phosphorescence lifetimes in the lower nanosecond (< 10 ns) range, accompanied by Stokes shifts of 4075 and 3250 cm^−1^ for **A** and **B**, respectively (Figure 1). The ^3^MLCT state of **B** was further shown to unlock reactions with aryl iodides to yield Bi(III) compounds [28, 30].

In accord with El Sayed's rule, and the distance dependence of the heavy atom effect [27, 28, 29, 30, 31], MLCT transitions can accelerate ISC in Bi compounds. A recent example is the compound Bi(bzq)_3_ (bzq = benzo[*h*]quinoline) of Marshak and co‐workers, which exhibits emission at 520 nm with *Φ* = 10(7)% at 25 °C following excitation into an MLCT state (Figure 1) [32]. The alternative strategy of encoding ligand‐to‐metal charge transfer (LMCT) into a Bi(III) compound to induce ISC has given much inferior results so far. Thus, Bi(III) compounds with tridentate NNN ligands have been restricted to provide phosphorescence emission in solvent glasses at cryogenic temperatures [33, 34, 35], or to emission solely in the solid state [36, 37].

Our group reported on the synthesis and reactivity of T‐shaped phosphorus and bismuth trisamides conformationally constrained by redox‐active NNN pincer ligands [38, 39, 40, 41, 42]. These *C*_2v_ symmetric molecules differ in their spectroscopic properties and reactivity from more common congeners with similar donor sets, but with *C*_3v_ symmetric pyramidal geometries at the central atom [43]. The geometric perturbation toward a T‐shape furnishes a low‐lying LUMO with Pn(p)‐orbital (Pn = pnictogen) character to be present at the pnictogen center which induces ambiphilic reactivity for phosphorus as well as for bismuth [44]. T‐shaped Bi(III) trisamides have received considerable attention recently since the combination of planarized Bi with redox‐active NNN pincer ligands unlocks transition metallomimetic properties [43, 44, 45, 46, 47, 48, 49, 50, 51, 52, 53]. However, their photophysical properties remain unexplored but hold large promise for NIR emission due to their small HOMO/LUMO gaps and intense colors in solution [43], In addition, the use of rigid ligands might suppress nonradiative decay typically observed for NIR emitters due to the singlet ground and excited triplet state being close in energy (energy gap law) [4, 5, 6, 7, 8, 9, 10, 11, 12, 13, 14, 15, 16, 17, 18, 19, 20, 21, 22, 23, 24, 25, 26, 27, 28, 29, 30, 31, 32, 33, 34, 35, 36, 37, 38, 39, 40, 41, 42, 43, 44, 45, 46, 47, 48, 49, 50, 51, 52, 53, 54]. Herein, we report on the photophysical properties of isostructural T‐shaped antimony and bismuth trisamides, both of which are emissive in the highly desirable NIR‐II region (1000–1700 nm) [6], in frozen solution as well as at 25 °C. In contrast to complex synthesis routes and/or utilization of precious metals as typically encountered in state‐of‐the‐art NIR emitters, both trisamides can be easily synthesized in high yields from simple precursors. This represents the first example of Sb(III) and Bi(III) compounds exhibiting NIR emission under these conditions, suggesting great potential for future developments for NIR emitters based on heavy main group elements.

**FIGURE 2:**
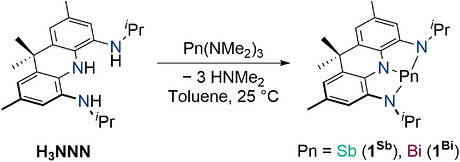
Synthesis of **1^Sb^** and **1^Bi^** *via* reaction of **H_3_NNN** with Pn(NMe_2_)_3_.

The recently reported Bi trisamide **1^Bi^** was isolated in the form of dark blue crystals (82%) upon reaction of the proligand **H_3_NNN** with Bi(NMe_2_)_3_ in toluene, followed by hexane extraction and recrystallization at −80°C [41, 55]. Analogously, dark green crystals of **1^Sb^** were obtained in 73% isolated yield utilizing Sb(NMe_2_)_3_ as the pnictogen source (Figure 2). Both compounds are highly sensitive toward trace moisture and oxygen [41].

**FIGURE 3:**
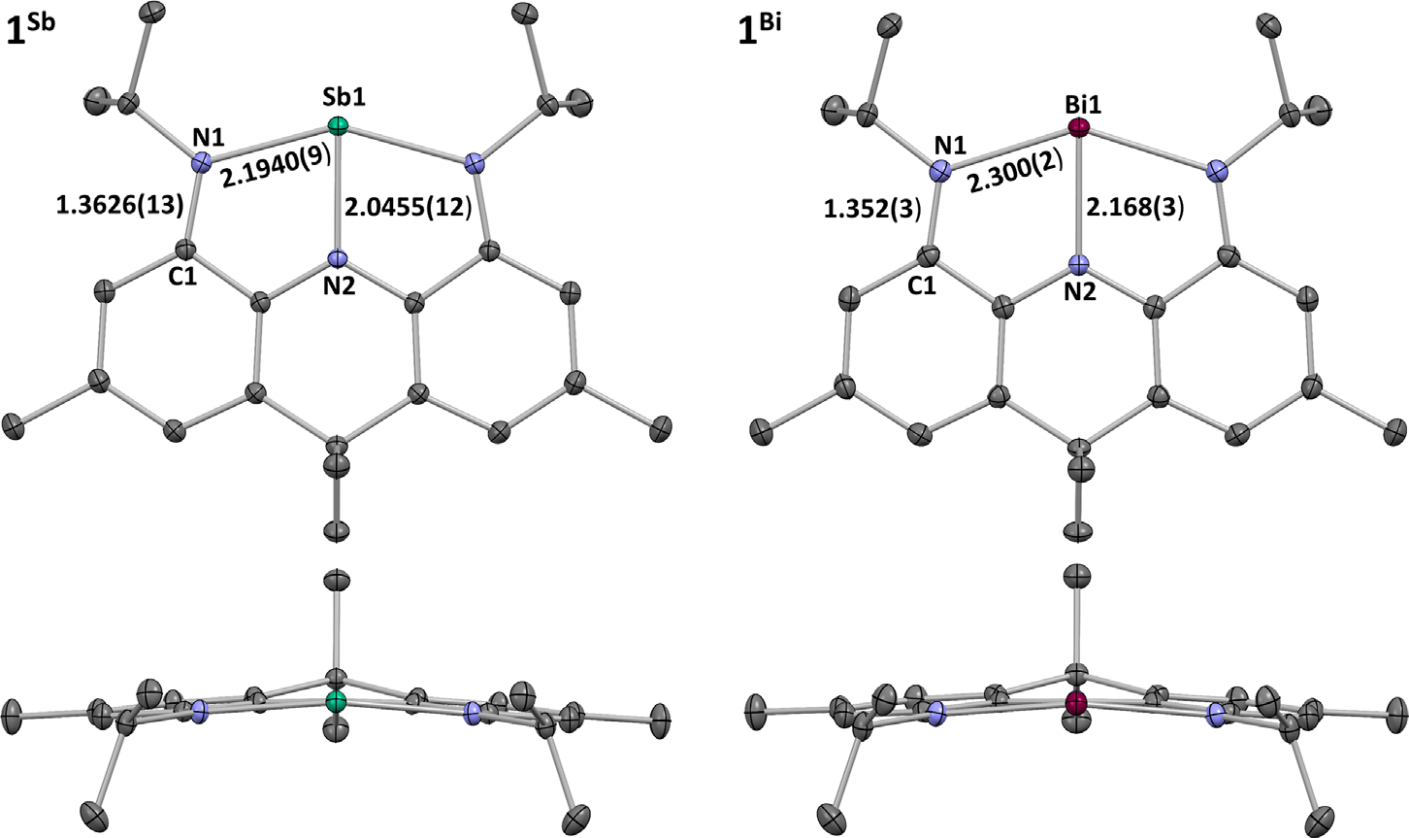
Molecular structures of **1^Sb^** and **1^Bi^** in the solid state derived by scXRD, hydrogen atoms were omitted for clarity, bond lengths in Å.

The molecular structure in the solid state, as obtained by single crystal X‐ray diffraction (scXRD), confirms the expected T‐shaped structure of **1** ^**Sb**^ (Figure 3). As expected, the Sb–N bond lengths (Sb1–N1: 2.1940(9) Å; Sb1–N2: 2.0455(12) Å) fall between those of the phosphorus analogue **1** ^**P**^[38] (P1–N1: 1.8601(14) Å; P1–N2: 1.710(2) Å)  and **1** ^**Bi**^[41] (Bi1–N1: 2.300(2) Å; Bi1–N2: 2.168(3) Å). Electron density transfer from the redox‐active ligand to the planarized pnictogen gives rise to pronounced quinone character within the acridane pincer scaffold (see Supporting Information). This is reflected in the comparably short N1–C1 bond distances which steadily decrease from the phosphorus to the bismuth analogues (N1–C2: 1.370(2) Å (**1** ^**P**^), 1.3626(13) Å (**1** ^**Sb**^), 1.352(3) Å (**1** ^**Bi**^)), in line with gradually increasing electron transfer from the ligand to the pnictogen center [56]. In contrast to geometrically constrained Sb and Bi trisamides with more flexible diphenylamine derived NNN ligand scaffolds reported by Chitnis and co‐workers [47, 48, 49, 50, 51, 52], no indications for dimerization were observed in solution or in the solid state which we ascribe to the higher rigidity of our pincer ligand (Figures S7–S12). The shortest Pn–Pn distance in the solid state (**1** ^**Sb**^: 3.79 Å / **1** ^**Bi**^: 3.87 Å) is close to the sum of the respective van‐der‐Wals radii (**1** ^**Sb**^: 4.12 Å / **1** ^**Bi**^: 4.14 Å) [31], and longer than the sum of their respective single‐bond radii (**1** ^**Sb**^: 2.80 Å / **1** ^**Bi**^: 3.02 Å) [57].

**FIGURE 4:**
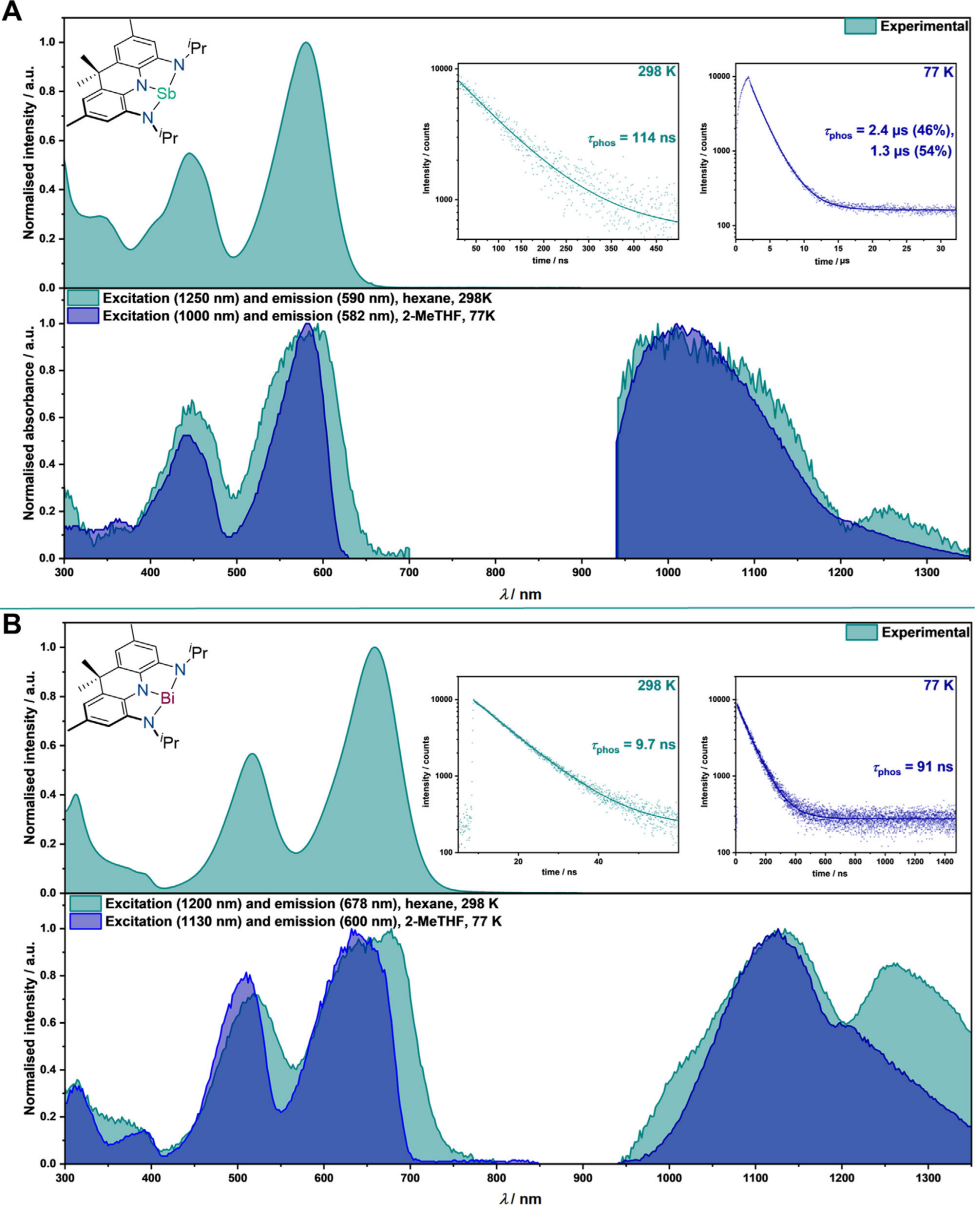
Absorbance, excitation, and emission spectra of **1** ^**Sb**^ (A) and **1** ^**Bi**^ (B) in different solvents and temperatures; Insets: Emission lifetimes at 298 and 77 K.

We began our investigations focusing on **1** **^Bi^**, inspired by the recent studies on T‐shaped bismuthinidenes [28, 29]. **1** **^Bi^** features two intense absorption bands in the visible range at 25 °C, which are located at *λ* = 658 and 516 nm in *n*‐hexane. Upon excitation into any of these bands, **1** **^Bi^** emits in the NIR‐II region with an emission maximum of *λ*_phos_ = 1134 nm and a second maximum at *λ*_phos_ = 1260 nm (Figure 4). The latter is likely attributable to relaxation to a higher vibronic level of the S_0_ ground state, consistent with a spacing of Δ$\tilde{\nu }$ = 882 cm^−1^. Excitation spectra recorded at the respective wavelengths compare well with the absorption spectrum, indicating that emissive deactivation operates *via* a single low‐lying excited state, regardless of the applied excitation wavelength in accordance with Kasha's rule [27, 58]. Excitation spectra recorded in 50 nm steps from 1000 to 1350 nm confirm that the emission stems from only one species (Figure S18). Concentration‐dependent photoluminescence and UV‐Vis absorptive measurements, moreover, reveal no differences in emission behavior across a range of 2.5–50 µM (Figures S13–S17). Additionally, emission decay traces recorded at 50 nm steps are monoexponential and provide identical lifetimes from *λ*_det_ = 1000 to 1350 nm (Figure S19 and Table S3). The measured lifetime of *τ*_phos_ = 9.7 ns is suggestive of considerable nonradiative quenching effects, despite the ligand's rigidity, and the value measured for the absolute quantum yield of *Φ*_phos_ ≤ 10^−5^ supports this assignment. These values compare favorably to recently reported Bi(I) compounds **A** and **B** that exhibit similar lifetimes and quantum yields (Figure 1) [28, 29]. At cryogenic temperatures in 2‐MeTHF, the emission is shifted hypsochromically such that the maximum lies at *λ*_phos_ = 1126 nm with a shoulder at 1205 nm. In agreement with the assumption that quenching effects at 25 °C shorten the emission lifetime, the lifetime at cryogenic temperatures is extended to *τ*_phos_ = 91.3 ns. Due to the large differences between the excitation and emission maximum of Δ*E_em–exc_* = 6380 cm^−1^ at 77 K and Δ*E*_em–exc_ = 6150 cm^−1^ at 25 °C, the profound similarity to the emission of **A** and **B**, as well as the excellent match to the calculated phosphorescence emission for **1** ^**Bi**^ (see below), it is assumed that this emission stems from an excited triplet state and is thus phosphorescence. The larger Stokes shift obtained for **1** ^**Bi**^ when compared to T‐shaped Bi(I) systems reported previously by the Cornella and Winter groups indicates a larger energy gap between the S_1_ and the T_1_ state in the case of **1** ^**Bi**^ compared to the pincer‐type bismuthinidenes **A** and **B**.

**1** ^**Sb**^ also features two intense absorption maxima in the visible region at *λ  =* 580 and 445 nm (with a shoulder at ∼460 nm) which are blue‐shifted by Δ*E* = 2040 and 3090 cm^−1^ when compared to isostructural **1** ^**Bi**^. In contrast to **1** ^**Bi**^, the S_0_→S_2_ transition does not feature a Gaussian line‐profile, which is indicative of multiple absorption processes for **1** ^**Sb**^ being operative in this region (Figure 4). **1** ^**Sb**^ is weakly fluorescent at *λ*_flu_ = 666 nm at 298 K and at *λ*_flu_ = 653 nm at 77 K (Figures S26, S33–S35). Although this fluorescence is very weak, it is detectable with the naked eye, leading to an appealing red shimmer of concentrated solutions of **1** ^**Sb**^ in certain light sources. While the intensity of this emission is too weak at *Φ*_flu_ = 0.03% to accurately determine lifetimes, it lies in the range of ns (Figure S34). **1** ^**Sb**^ also phosphoresces in the NIR‐II region in both hexane solution at 25 °C and in 2‐MeTHF at 77 K. The emission of **1** ^**Sb**^ is blue‐shifted compared to **1** ^**Bi**^, to *λ*_phos_ *=* 1010 nm and at *λ*_phos_ = 988 nm for 25 °C and cryogenic temperatures, respectively. Emission decay traces, excitation spectra, and concentration‐dependent measurements (Figures S23–S30) are consistent across the entire range of the detection window and concentration range. The measured lifetimes are considerably longer than those found for **1** ^**Bi**^ with *τ*_phos_ = 113.8 ns at 25 °C and *τ*_phos,1_ = 2.4 µs (46%) and *τ*_phos,2_ = 1.3 µs (54%) at 77 K. The longer phosphorescence lifetimes are attributed to both the larger energy gap Δ*E*_T1‐S0_ between the T_1_ state and the S_0_ ground state (Δ*E*_em‐exc_ = 6940 cm^−1^ and Δ*E*_em‐exc_ = 6150 cm^−1^ at 25 °C and cryogenic temperatures, respectively) and the smaller spin‐orbit coupling constant of Sb compared to Bi [59]. As for its bismuth congener, the quantum yield at 25 °C lies outside the range of the device's accuracy with *Φ*_phos_ ≤ 10^−5^. To the best of our knowledge, this is the first account of an NIR emission at 25 °C in solution by a molecular Sb compound.

**FIGURE 5:**
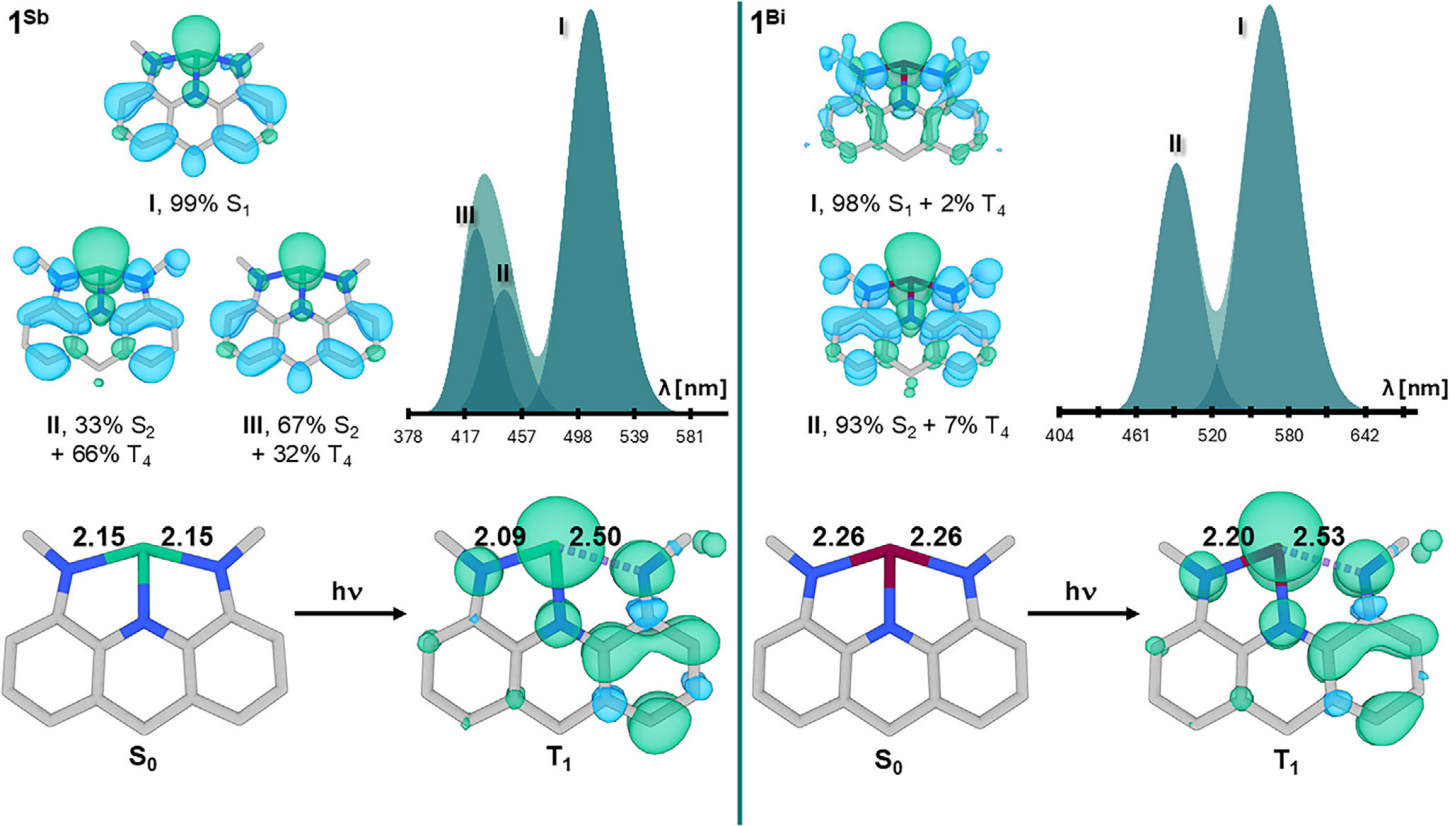
Calculated difference densities and structural changes upon excitation of **1** ^**Sb**^ (left) and **1** ^**Bi**^ (right), see ESI for computational details, bond lengths in Å.

We aimed to gain deeper insight into the photophysical properties of both T‐shaped trisamides by theoretical calculations. CASSCF calculations on truncated models of **1** ^**Sb**^ and **1** ^**Bi**^ were employed to establish a suitable description of the electronic structure of both Pn(NNN) compounds to describe their photophysical properties. The proper description of the redox‐state of planarized Bi NNN pincer compounds in their ground‐state has been debated in the past with Bi(I) and Bi(III) oxidation states or biradicaloid character being proposed [49, 50, 51, 52, 53, 54, 55, 56, 57, 58, 59, 60, 61]. Consistent with our earlier spectroscopic and computational studies that favor a Pn(III) redox state, pronounced electron delocalization between the redox‐active ligand and the T‐shaped Pn(III) centers results in polarized, yet highly covalent Pn═N π‐bonds between the pnictogen and the central nitrogen atom of the pincer ligand [39, 40, 41, 42]. Despite this underlying complexity, the CASSCF results demonstrate that the wavefunction can be represented by a single‐determinant method for both trisamides (see Supporting Information). **1** ^**Sb**^ and **1** ^**Bi**^ exhibit S_0_→S_1_ LMCT transitions resulting from an excitation from a ligand‐centered HOMO to a ligand‐pnictogen π*‐antibonding LUMO. When trying to reproduce the experimental absorption spectrum of **1** ^**Sb**^ in the S_0_→S_2_ region, TD‐DFT predicts only one transition to be present, which disagrees with the pronounced shoulder observed experimentally (Figure 4). This changes when introducing spin‐orbit coupling, which leads to mixing of the S_2_ state with the energetically proximate T_4_ state producing two separate bands in full agreement with the experimental absorption spectrum. In contrast, the energetic separation of the corresponding states in **1** ^**Bi**^ is larger, and state‐mixing has a negligible influence on the S_0_→S_2_ transition (Figure 5). Nevertheless, the overall nature of the S_0_→S_2_ transitions in both compounds is similar, namely a Pn(p)–ligand π→π* excitation best described as HOMO–1 to LUMO transitions. As the latter is strongly pnictogen‐centered, an overall LMCT character ensues. The T_1_ states of both trisamides were subsequently optimized. In both cases, a distorted geometry was obtained in which the pnictogen atom features two markedly different flanking Pn–N bond lengths. The corresponding spin‐densities are primarily localized at the pnictogen atoms and the arene bound to the weakly interacting nitrogen atom, consistent with a biradical description (Figure 5). The calculated emission energies are in full agreement with the Stokes shift observed experimentally. Consequently, we ascribe the large Stokes shifts observed for **1** ^**Sb**^ and **1** ^**Bi**^ to a significant structural reorganization during photoexcitation and pronounced charge separation within the emissive T_1_ state.

In summary, we present the first examples of Sb(III) and Bi(III) compounds that exhibit NIR‐II phosphorescence in solution at 25 °C. Visible‐light‐induced ligand‐to‐metal charge transfer leads to large Stokes shifts and long‐lived triplet states, with **1** ^**Sb**^ showing lifetimes up to the microsecond range. These findings not only challenge the dominance of precious metal‐based NIR emitters but also establish a new design paradigm for photofunctional materials based on main group elements. This study opens exciting avenues for future photochemical bond activation and optoelectronic applications by heavy pnictogen compounds.

## Conflicts of Interest

The authors declare no conflicts of interest.

## Supporting information



The authors have cited additional references within the Supporting Information [62, 63, 64, 65, 66, 67, 68, 69, 70, 71, 72, 73, 74, 75, 76].
