# Supplementary material for: Phosphorescence of Heavy, T‐Shaped Pnictogen Trisamides in Solution at Room Temperature in the Near‐Infrared II Region
Source: Chemistry. 2026 May 1;32(27):e71049. doi: 10.1002/chem.71049 (PMC13380389; doi:10.1002/chem.71049)
Supplement: Supplementary file 1 — The authors have cited additional references within the Supporting Information [62, 63, 64, 65, 66, 67, 68, 69, 70, 71, 72, 73, 74, 75, 76]. [file CHEM-32-e71049-s001.docx]

*Electronic Supporting Information*

Phosphorescence of Heavy, T-shaped Pnictogen Trisamides in Solution at Room Temperature in the Near-Infrared II Region

Katharina L. Deuter,^[a],§^ Sotirios Pavlidis,^[b],§^ Rainer F. Winter,^[a],^* Peter Coburger^[c],^* and Josh Abbenseth^[b],[d],^*

[a] K. L. Deuter, Prof. R. F. Winter
Faculty for Chemistry
University of Konstanz
Universitätsstraße 10, 78457, Konstanz, Germany
E-mail: [rainer.winter@uni-konstanz.de](mailto:rainer.winter@uni-konstanz.de)

[b] S. Pavlidis, Dr. J. Abbenseth
Institut für Chemie
Humboldt-Universität zu Berlin
Brook-Taylor-Str. 2, 12489 Berlin, Germany

[c] Dr. P. Coburger
Department of Inorganic Chemistry
TU München
Lichtenbergstraße 4, 85747 Garching, Germany
E-mail: [peter.coburger@tum.de](mailto:peter.coburger@tum.de)

[d] Dr. J. Abbenseth
Department of Chemistry
University of Manchester
Oxford Road, Manchester M13 9PL, U.K.
E-mail: [josh.abbenseth@manchester.ac.uk](mailto:josh.abbenseth@manchester.ac.uk)

Table of Contents

[Materials and Methods S3](#_Toc210476282)

[Synthetic procedures and characterization S4](#_Toc210476283)

[Synthesis of **1^Sb^** S4](#_Toc210476284)

[New synthesis of **1^Bi^** S8](#_Toc210476285)

[Single crystal X-ray crystallography S10](#_Toc210476286)

[Photoluminescence Spectroscopy S18](#_Toc210476287)

[Density functional theory calculations S32](#_Toc210476288)

[References S38](#_Toc210476289)

# Materials and Methods

**NMR spectra** were recorded on AVANCE II 300 MHz, Bruker Avance 400 MHz and Bruker Avance III 500 MHz NMR spectrometers. Chemical shifts are referenced to the signal of residual protonated solvent.

**IR spectra** were recorded on a Bruker ALPHA spectrometer with an ATR sampling unit.

**Elemental analyses** were performed with a HEKA Euro 3000EA elemental analyzer.

**Photoluminescence Spectroscopy** Steady state photoluminescence spectra were recorded with the FluoTime 300 spectrometer from *PicoQuant*. All measurements were performed on solutions of the complexes in dry 2-MeTHF, *n*-hexane, CH_2_Cl_2_ or benzene under a nitrogen atmosphere. Measurements were conducted on solutions with concentrations ranging from 1.25 µM to 40 µM. Solutions in 2-MeTHF were handled in 7 mm quartz tubes purchased from *PicoQuant*. Spectra in *n*-hexane at r.t. were recorded using a custom‑built quartz cuvette with a vacuum valve, whereby the original cuvette was a 1 cm cell with a 221.01Qs tube purchased from *Hellma Analytics*. UV-vis absorption spectra were recorded on a TIDAS fiber optic diode array spectrometer, consisting of a combination of MCS UV/Vis and PGS NIR instruments from *j&m Analytic* AG prior to and after measurements and subsequently compared to guarantee that no degradation had occurred during the measurement. Lifetimes were recorded using the FluoTime 300 spectrometer from *PicoQuant.*

All experiments with air-sensitive compounds were carried out in a glovebox or in a fume hood employing Schlenk techniques under a dry Ar atmosphere. Traces of water and oxygen were removed *via* heating of glassware under vacuum prior to use.

All solvents except THF were dried and degassed by a MBraun solvent purification system. THF was dried over sodium, distilled and stored over molecular sieves. Deuterated solvents and 2-MeTHF were degassed *via* three freeze-pump-thaw cycles and stored over molecular sieves.

All commercially available chemicals were used without purification unless otherwise noted. H_3_NNN, Bi(NMe_2_)_3_ and Sb(NMe_2_)_3_ were prepared according to literature procedures.^[1-3]^

# Synthetic procedures and characterization

## Synthesis of 1^Sb^

**H_3_NNN** (100 mg, 285 µmol, 1.00 eq.) is dissolved in toluene (10 mL) and added to a Schlenk flask containing Sb(NMe_2_)_3_ (80.0 mg, 313 µmol, 1.10 eq.) dissolved in toluene (5 mL) under an inert gas atmosphere. The dark green reaction solution is stirred for two days at 25 °C, followed by removal of the solvent *in vacuo*, extraction with hexane (3 x 5 mL), concentration and storing the flask at −80 °C for three days. **1^Sb^** is obtained as a fine dark green crystals after filtration and drying *in vacuo* (98.2 mg, 208.4 µmol, 73%).

NMR: (C_6_D_6_, 25 °C) ^1^H NMR (500 MHz) *δ* (ppm) = 6.98 (d, ^4^*J*_H–H_ = 1.6 Hz, 2H, C^Ar^*H*), 6.92 – 6.89 (m, 2H, C^Ar^*H*), 4.66 (sep, ^3^*J*_H–H_ = 6.5 Hz, 2H, NC*H*(CH_3_)_2_), 2.48 (s, 6H, C^Ar^C*H*_3_), 1.82 (s, 6H, C^quart^(C*H*_3_)_2_), 1.58 (d, ^3^*J*_H–H_ = 6.5 Hz, 12H, NCH(C*H*_3_)_2_).

^13^C{^1^H} NMR (126 MHz) *δ* (ppm) = 144.72 (C^Ar^), 134.13 (C^Ar^), 132.90 (C^Ar^), 131.52 (C^Ar^), 115.35 (*C*^Ar^H), 108.86 (*C*^Ar^H), 50.64 (*C*H(CH_3_)_2_), 37.79 (*C*^quart^(CH_3_)_2_), 35.92 (C^quart^(*C*H_3_)_2_), 26.54 (NCH(*C*H_3_)_2_), 22.48 (C^Ar^*C*H_3_).

ATR-IR (solid): *ṽ* (cm^−1^) = 2952, 1584, 1305, 1135, 821.

Elem. Anal. found (calcd) for (C_44_H_58_SbN_3_): C, 58.82 (58.74); H, 6.46 (6.43); N, 8.66 (8.94).


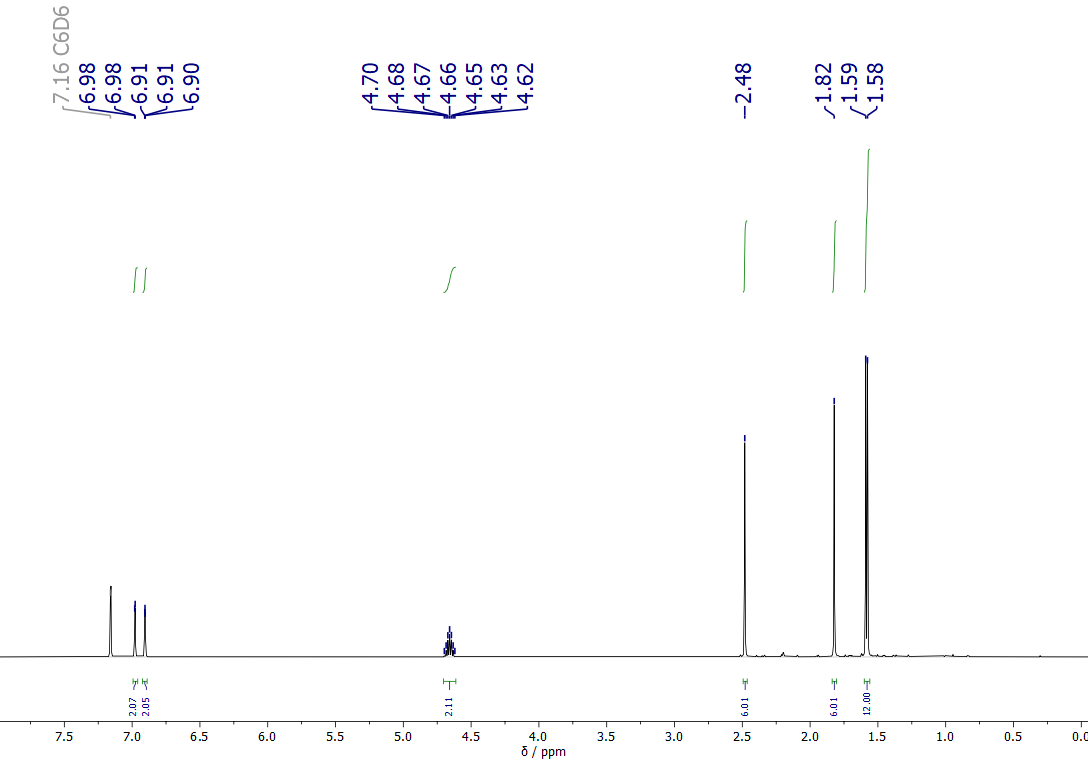


**Figure S1.** ^1^H NMR spectrum of **1^Sb^**, C_6_D_6_, 25 °C.


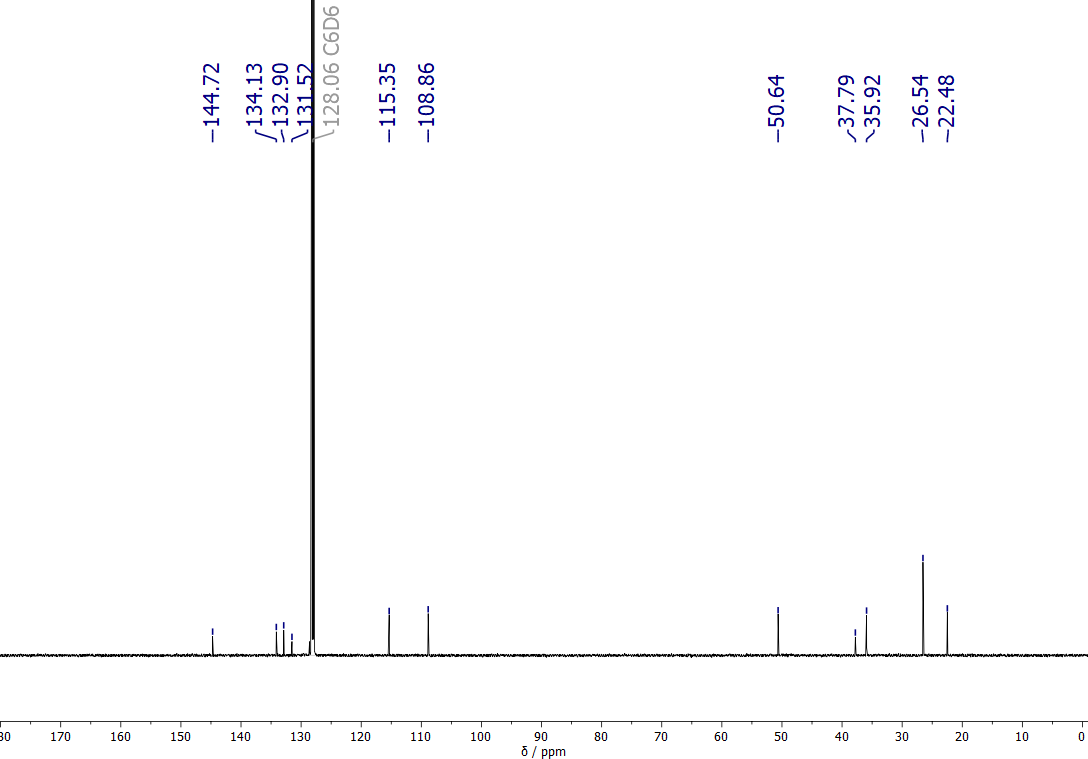


Figure S2. ^13^C{^1^H} NMR spectrum of 1^Sb^, C_6_D_6_, 25 °C.


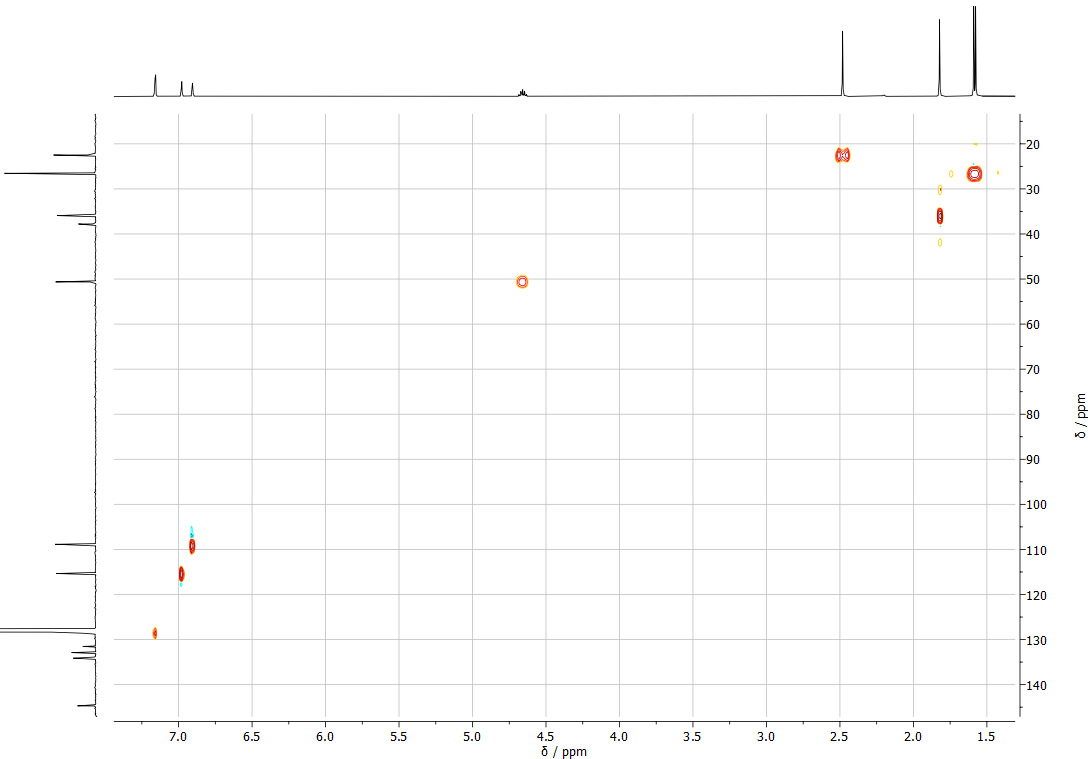


Figure S3. HSQC NMR spectrum of 1^Sb^, C_6_D_6_, 25 °C.


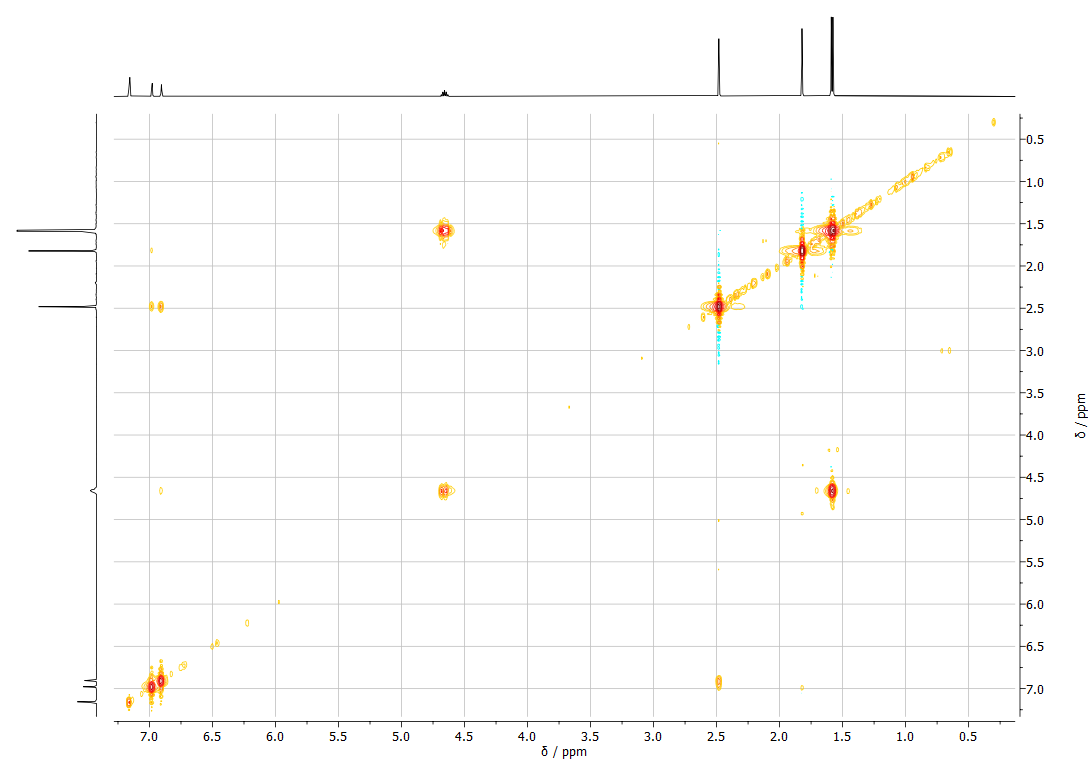


Figure S4. COSY NMR spectrum of 1^Sb^, C_6_D_6_, 25 °C.


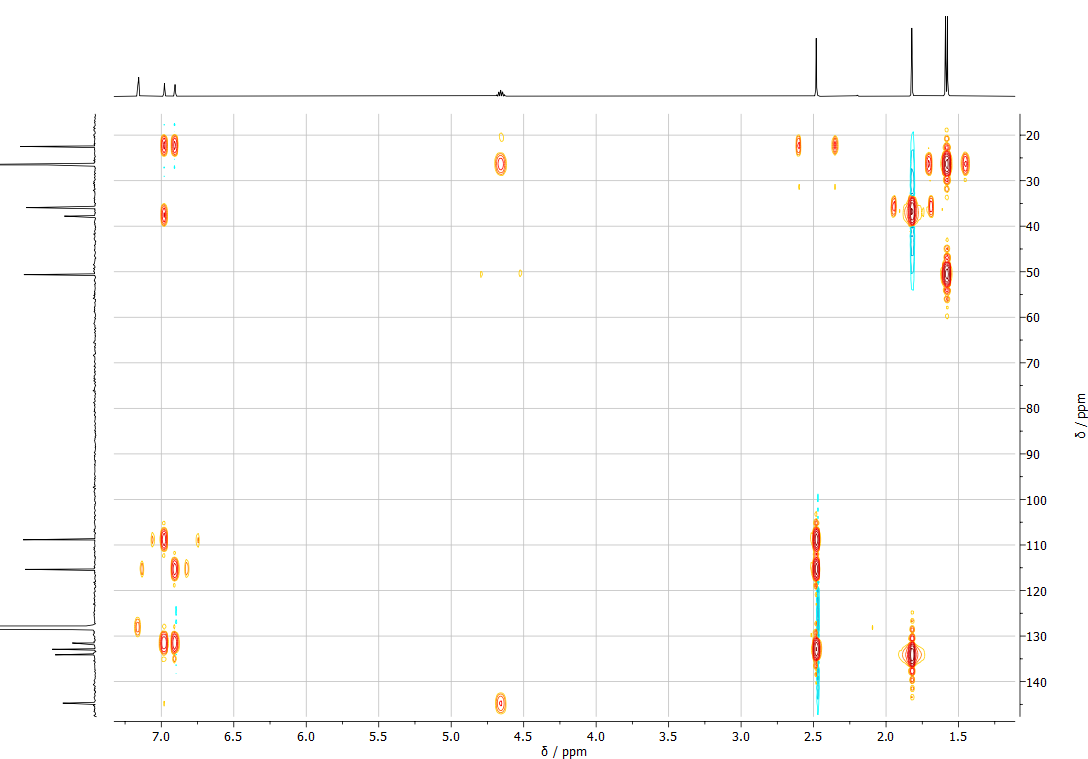


Figure S5. HMBC NMR spectrum of 1^Sb^, C_6_D_6_, 25 °C.


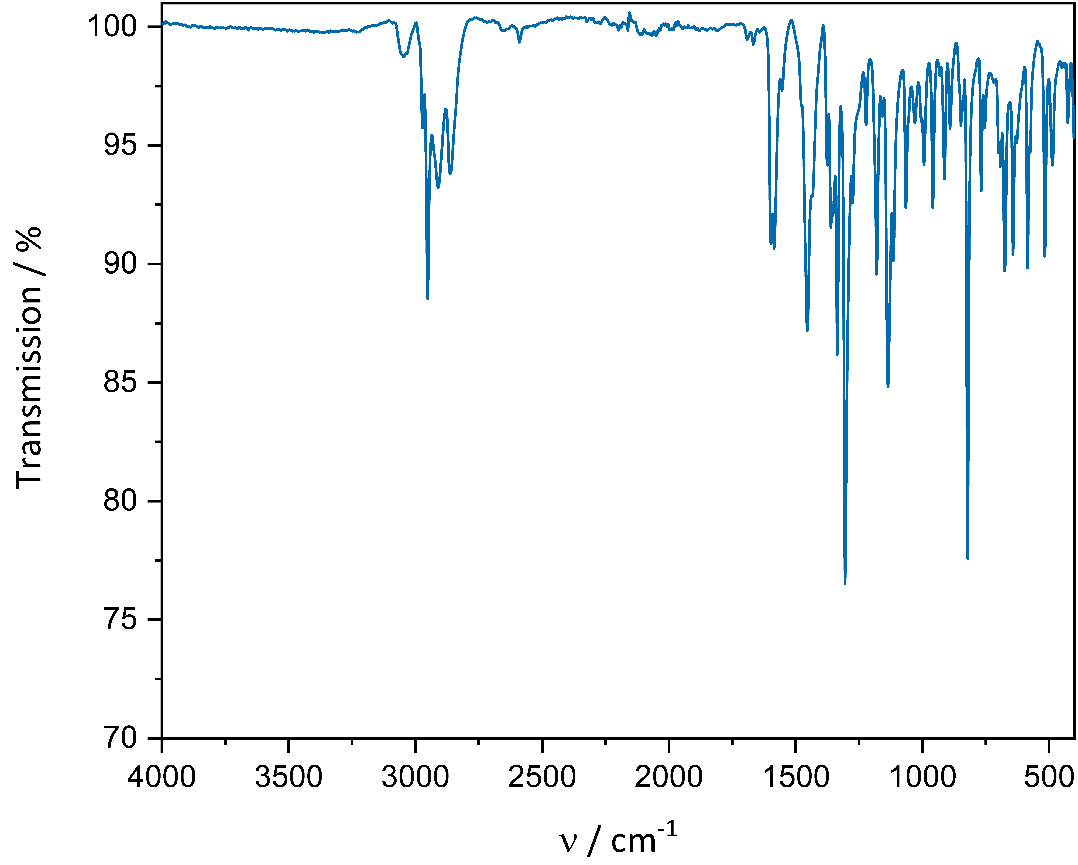


Figure S6. ATR-IR spectrum of 1^Sb^, solid, 25 °C.

## New synthesis of 1^Bi^

**1^Bi^** was synthesized according to a slightly modified literature procedure.^[4]^ **H_3_NNN** (187.9 mg, 535 µmol, 1.00 eq.) is dissolved in toluene (15 mL) and added to a Schlenk flask containing Bi(NMe_2_)_3_ (192 mg, 563 µmol, 1.05 eq.) dissolved in toluene (5 mL). The dark blue reaction solution is stirred for 30 min at 25 °C, followed by removal of the solvent *in vacuo*, extraction with hexane (2 x 15 mL), concentration and storing the flask at −80 °C for three days. **1^Bi^** is obtained as dark blue crystals after filtration and drying *in vacuo* (243.6 mg, 437 µmol, 82%). NMR spectroscopy was in full agreement with previously reported data.

Elem. Anal. found (calcd) for (C_44_H_58_BiN_3_): C, 49.99 (49.55); H, 5.44 (5.42); N, 7.59 (7.54).

# Single crystal X-ray crystallography

A green, block-shaped crystal was mounted on a MiTeGen micromount with perfluoroether oil. Data for mo_ja25_ja_iii_97_2_a (**1^Sb^**) were collected from a shock-cooled single crystal at 100.00 K on a Bruker D8 VENTURE dual wavelength Mo/Cu three-circle diffractometer with a microfocus sealed X-ray tube using a mirror optics as monochromator and a Bruker PHOTON III detector. The diffractometer was equipped with an Oxford Cryostream 800 low temperature device and used Mo *K_α_* radiation (λ = 0.71073 Å). All data were integrated with SAINT and a multi-scan absorption correction using SADABS 2016/2 was applied.^[5,6]^ The structure was solved by direct methods with SHELXT 2018/2 and refined by full-matrix least-squares methods against *F*^2^ using SHELXL-2019/2.^[7,8]^ All non-hydrogen atoms were refined with anisotropic displacement parameters. All hydrogen atoms were refined isotropic on calculated positions using a riding model with their *U*_iso_ values constrained to 1.5 times the *U*_eq_ of their pivot atoms for terminal sp^3^ carbon atoms and 1.2 times for all other carbon atoms. Crystallographic data for the structures reported in this paper have been deposited with the Cambridge Crystallographic Data Centre.^[9]^ CCDC 2493257 contains the supplementary crystallographic data for this paper. These data can be obtained free of charge from The Cambridge Crystallographic Data Centre via www.ccdc.cam.ac.uk/structures. This report was generated using FinalCif.^[10]^

**Table S1** Crystal data and structure refinement for **1^Sb^**.

| CCDC number | 2493257 |
| --- | --- |
| Empirical formula | C_23_H_30_N_3_Sb |
| Formula weight | 470.25 |
| Temperature [K] | 100.00 |
| Crystal system | orthorhombic |
| Space group (number) | $Cmca$ (64) |
| *a* [Å] | 23.114(2) |
| *b* [Å] | 17.7437(17) |
| *c* [Å] | 10.0406(9) |
| α [°] | 90 |
| β [°] | 90 |
| γ [°] | 90 |
| Volume [Å^3^] | 4117.9(7) |
| *Z* | 8 |
| *ρ*_calc_ [gcm^−3^] | 1.517 |
| *μ* [mm^−1^] | 1.353 |
| *F*(000) | 1920 |
| Crystal size [mm^3^] | 0.020×0.050×0.150 |
| Crystal colour | green |
| Crystal shape | block |
| Radiation | Mo *K_α_* (λ=0.71073 Å) |
| 2θ range [°] | 4.98 to 66.44 (0.65 Å) |
| Index ranges | −35 ≤ h ≤ 35 −27 ≤ k ≤ 27 −15 ≤ l ≤ 15 |
| Reflections collected | 152113 |
| Independent reflections | 4042  *R*_int_ = 0.0521 *R*_sigma_ = 0.0127 |
| Completeness to  θ = 25.242° | 99.8 % |
| Data / Restraints / Parameters | 4042 / 1 / 135 |
| Absorption correction T_min_/T_max_ (method) | 0.6605 / 0.7465  (multi-scan) |
| Goodness-of-fit on *F*^2^ | 1.106 |
| Final *R* indexes  [*I*≥2σ(*I*)] | *R*_1_ = 0.0164 w*R*_2_ = 0.0410 |
| Final *R* indexes  [all data] | *R*_1_ = 0.0197 w*R*_2_ = 0.0436 |
| Largest peak/hole [eÅ^−3^] | 0.63/−0.44 |

**Table S1** Bond lengths and angles for **1^Sb^**.

| **Atom–Atom** |  | **Length [Å]** |
| --- | --- | --- |
| N1–C1 |  | 1.3626(13) |
| N1–C11 |  | 1.4670(13) |
| N1–Sb1 |  | 2.1940(9) |
| Sb1–N2 |  | 2.0455(12) |
| N2–C6^#1^ |  | 1.3761(11) |
| N2–C6 |  | 1.3762(11) |
| C1–C2 |  | 1.4159(13) |
| C1–C6 |  | 1.4213(13) |
| C6–C5 |  | 1.4047(13) |
| C7–C5 |  | 1.5264(12) |
| C7–C5^#1^ |  | 1.5264(12) |
| C7–C10 |  | 1.537(2) |
| C7–C9 |  | 1.547(2) |
| C8–C3 |  | 1.5089(15) |
| C11–C12 |  | 1.5250(15) |
| C11–C13 |  | 1.5317(16) |
| C2–C3 |  | 1.3859(14) |
| C3–C4 |  | 1.4067(15) |
| C4–C5 |  | 1.3877(14) |
|  |  |  |
| **Atom–Atom–Atom** |  | **Angle [°]** |
| C1–N1–C11 |  | 118.50(8) |
| C1–N1–Sb1 |  | 115.37(6) |
| C11–N1–Sb1 |  | 126.05(6) |
| N2–Sb1–N1 |  | 74.77(2) |
| N2–Sb1–N1^#1^ |  | 74.77(2) |
| N1–Sb1–N1^#1^ |  | 148.16(4) |
| C6^#1^–N2–C6 |  | 120.20(11) |
| C6^#1^–N2–Sb1 |  | 119.85(6) |
| C6–N2–Sb1 |  | 119.85(6) |
| N1–C1–C2 |  | 127.70(9) |
| N1–C1–C6 |  | 114.78(8) |
| C2–C1–C6 |  | 117.51(9) |
| N2–C6–C5 |  | 122.42(9) |
| N2–C6–C1 |  | 115.09(8) |
| C5–C6–C1 |  | 122.49(8) |
| C5–C7–C5^#1^ |  | 111.15(11) |
| C5–C7–C10 |  | 110.82(7) |
| C5^#1^–C7–C10 |  | 110.82(7) |
| C5–C7–C9 |  | 107.21(8) |
| C5^#1^–C7–C9 |  | 107.21(8) |
| C10–C7–C9 |  | 109.50(12) |
| N1–C11–C12 |  | 110.95(8) |
| N1–C11–C13 |  | 111.45(8) |
| C12–C11–C13 |  | 110.31(9) |
| C3–C2–C1 |  | 120.62(9) |
| C2–C3–C4 |  | 120.04(9) |
| C2–C3–C8 |  | 121.27(9) |
| C4–C3–C8 |  | 118.69(9) |
| C5–C4–C3 |  | 121.75(9) |
| C4–C5–C6 |  | 117.60(9) |
| C4–C5–C7 |  | 122.18(9) |
| C6–C5–C7 |  | 119.95(9) |

Bonds and angles to hydrogen atoms were omitted.

Symmetry transformations used to generate equivalent atoms:

#1: 1-X, +Y, +Z;


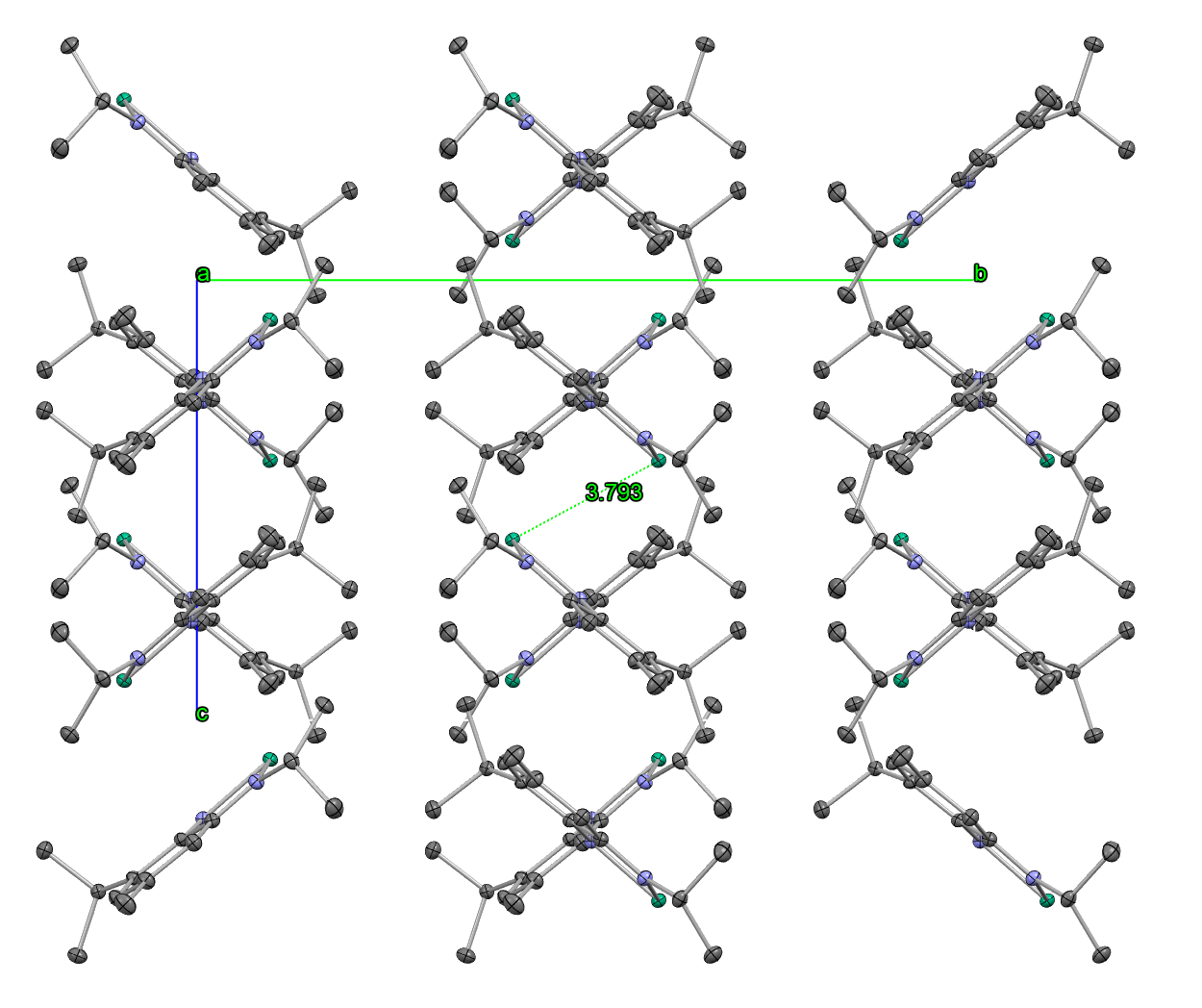


**Figure S7.** View of the molecular packing along the crystallographic a-axis of **1^Sb^**.


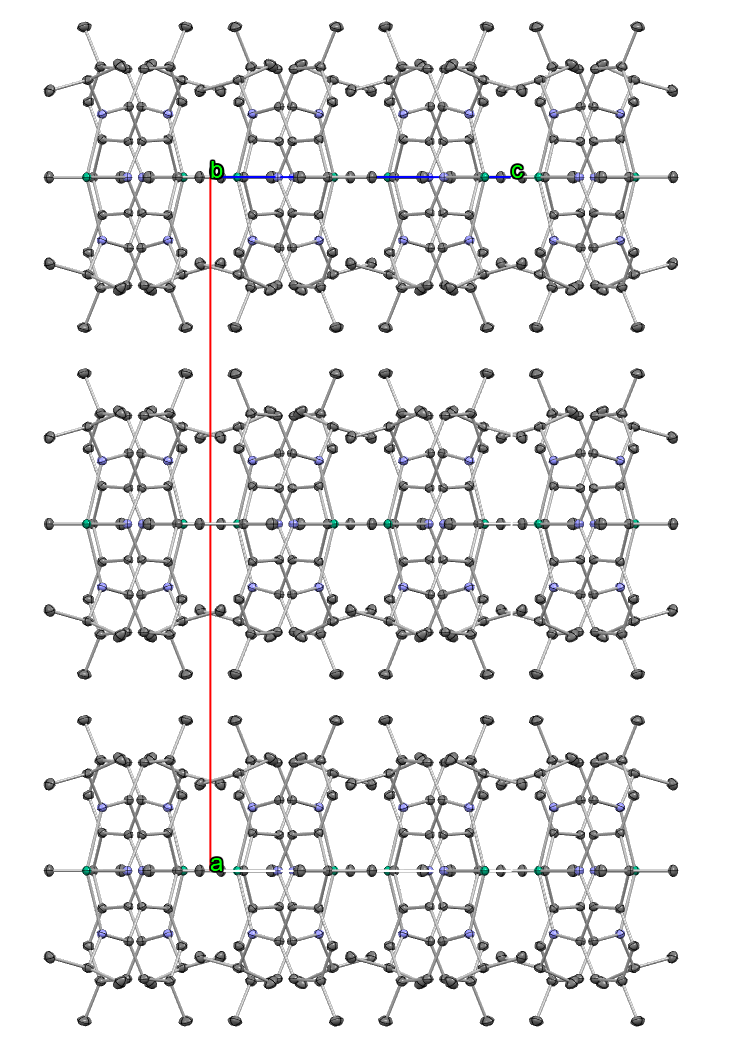
**Figure S8.** View of the molecular packing along the crystallographic b-axis of **1^Sb^**.

**Figure S9.** View of the molecular packing along the crystallographic c-axis of **1^Sb^**.


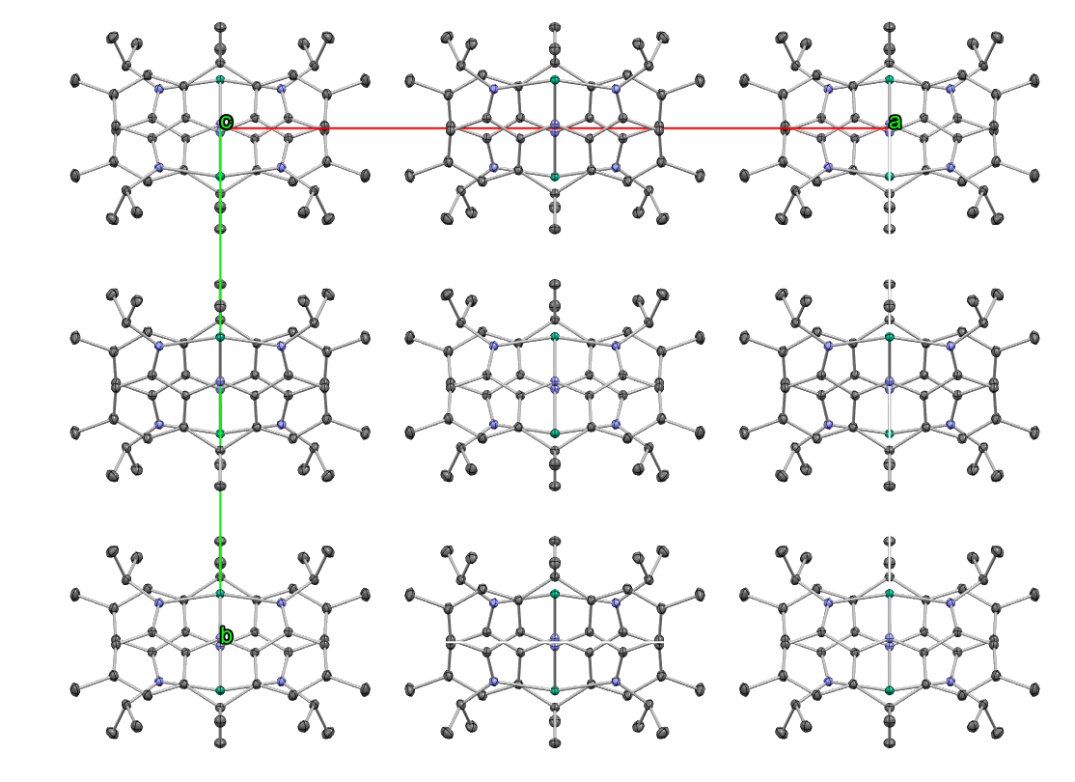


**
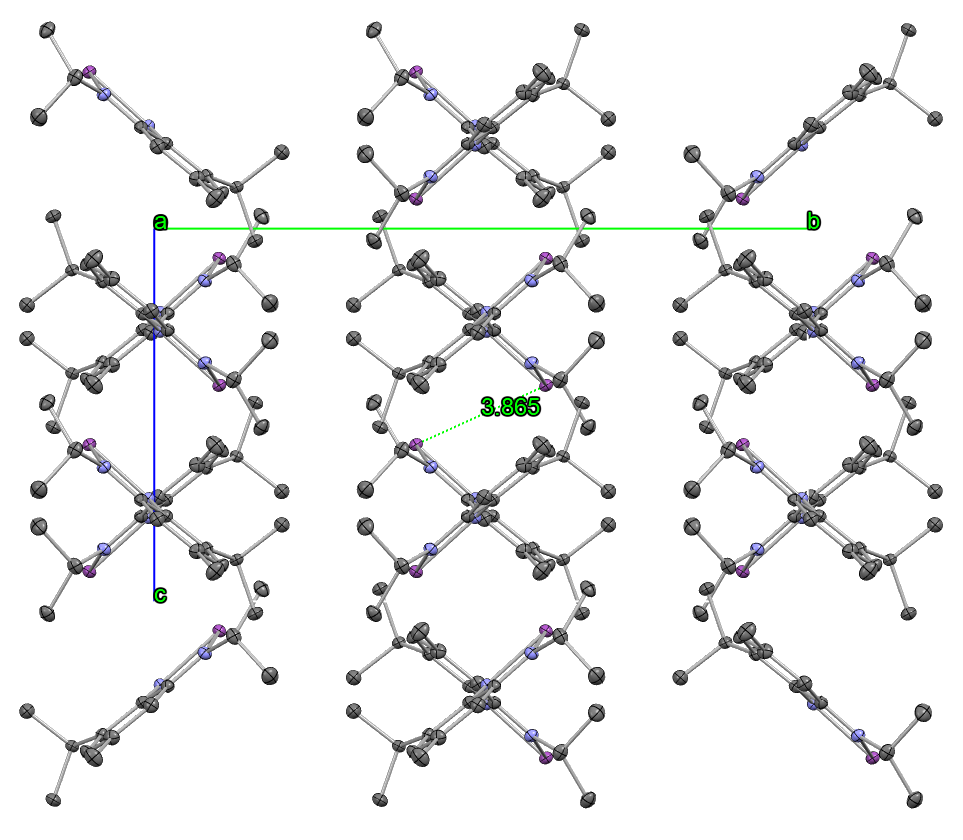
**

**Figure S10.** View of the molecular packing along the crystallographic a-axis of **1^Bi^**.


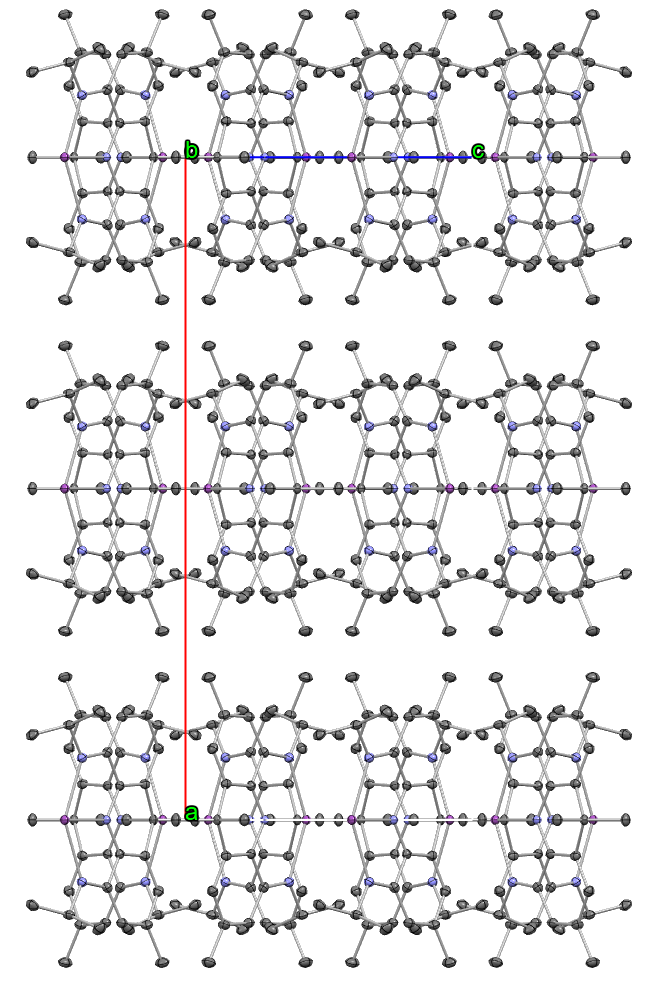


**Figure S11.** View of the molecular packing along the crystallographic b-axis of **1^Bi^**.


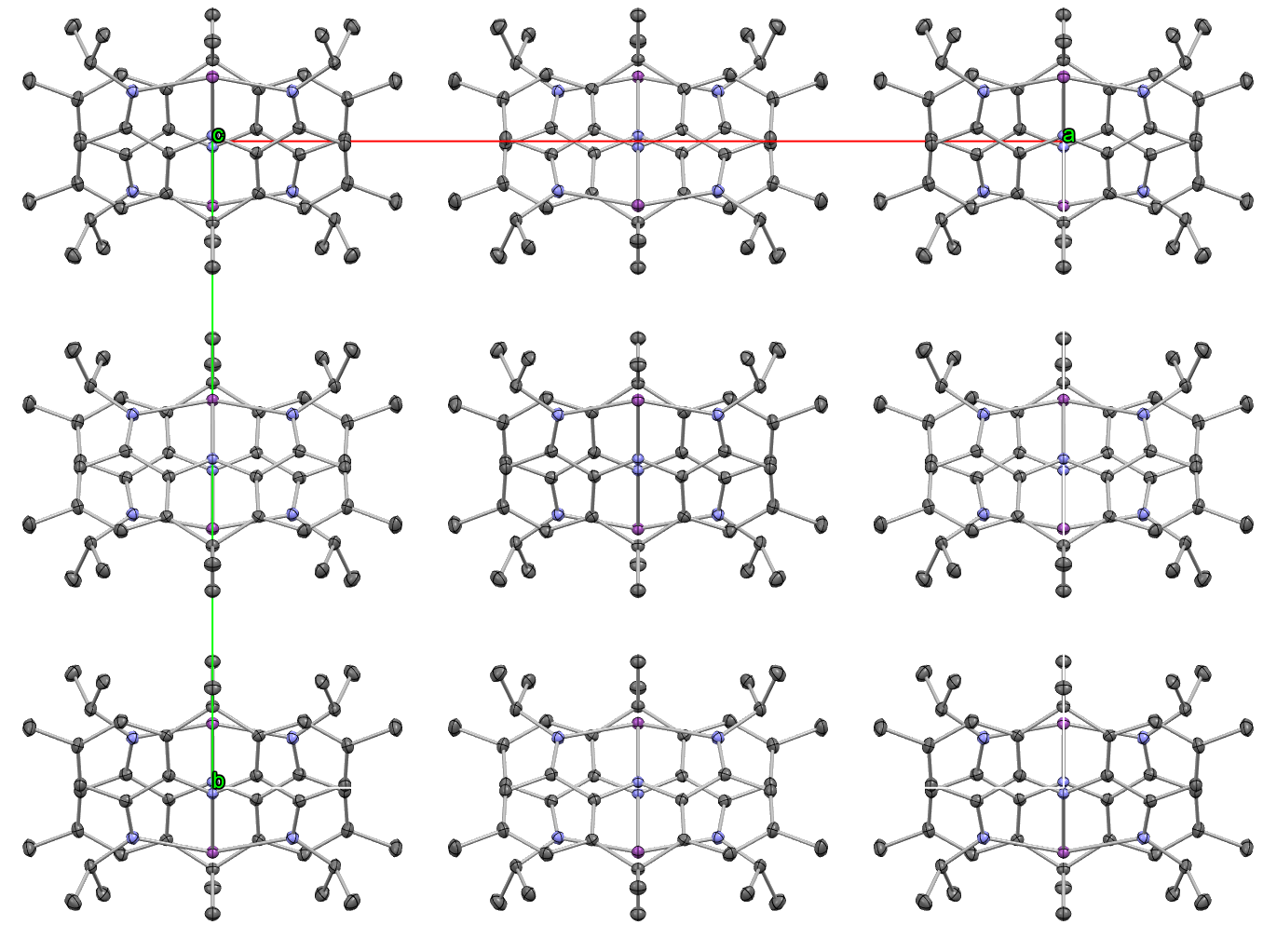


**Figure S12.** View of the molecular packing along the crystallographic c-axis of **1^Bi^**.

# Photoluminescence & UV-Vis Absorption Spectroscopy

Figure S13. UV-Vis absorption spectra of solutions of 1^Bi^ recorded in *n*-hexane at different concentrations at r.t.

Figure S14. UV-Vis absorption spectra of solutions of 1^Bi^ recorded in different solvents at r.t.

Figure S15. Emission and excitation spectra of 1^Bi^ in *n*-hexane and CH_2_Cl_2_ at r.t. and ca. c = 22 µM.

Figure S16. Emission and excitation spectra of 1^Bi^ in *n*-hexane at r.t. at differing concentrations.


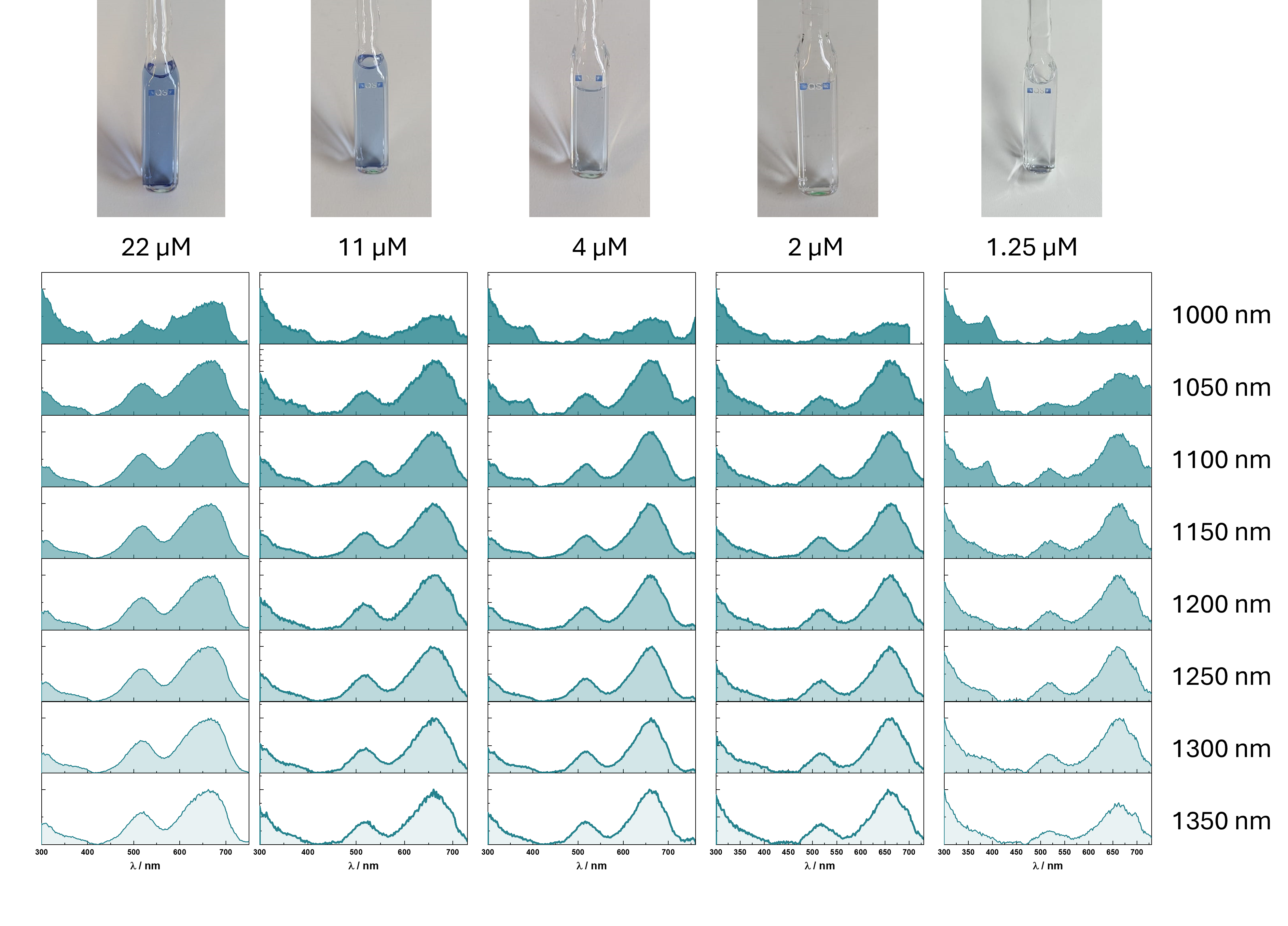
Figure S17. Normalized excitation spectra of the emission of 1^Bi^ detected at different wavelengths and concentrations in *n*-hexane at r.t.

Figure S18. Excitation spectra of the emission of 1^Bi^ recorded at different wavelengths in *n*‑hexane at r.t. c = 22 µM.

**Figure S19.** **Left:** Emission decay traces of the emission of **1^Bi^** recorded at different wavelengths in *n*‑hexane at r.t. c = 22 µM. **Right:** Intensity of the emission decay traces at *t* = 5.0 ns, and lifetimes extracted from emission decay traces at differing detection wavelengths.


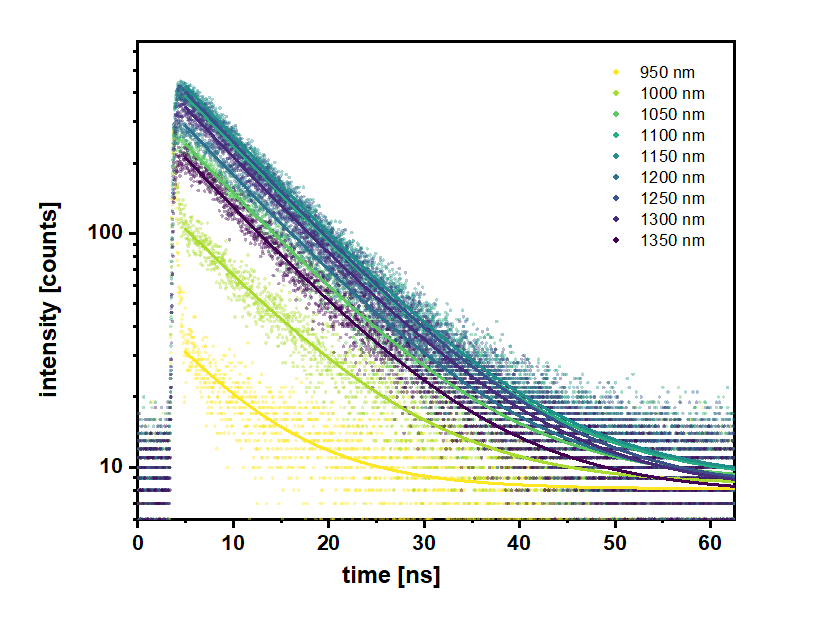


Figure S20. Emission decay traces and monoexponential fits of the emission of 1^Bi^ recorded at different wavelengths in *n*‑hexane at r.t. c = 22 µM.

| **λ_det_** |  | **y0** | | **A1** | | **t1** | | **k** | | **tau** | | **Statistics** | |
| --- | --- | --- | --- | --- | --- | --- | --- | --- | --- | --- | --- | --- | --- |
|  |  | Value | Standard Error | Value | Standard Error | Value | Standard Error | Value | Standard Error | Value | Standard Error | Reduced Chi-Sqr | Adj. R-Square |
| 950 |  | 8.08141 | 0.10759 | 42.40174 | 1.28014 | 8.20156 | 0.24222 | 0.12193 | 0.0036 | 5.68489 | 0.16789 | 11.66157 | 0.69971 |
| 1000 |  | 8.42028 | 0.1794 | 160.49324 | 1.4667 | 9.7967 | 0.10161 | 0.10208 | 0.00106 | 6.79055 | 0.07043 | 25.49806 | 0.95361 |
| 1050 |  | 8.65666 | 0.25357 | 384.79044 | 2.03205 | 9.88875 | 0.05976 | 0.10113 | 6.11126E-4 | 6.85436 | 0.04142 | 50.20554 | 0.98384 |
| 1100 |  | 8.62711 | 0.30079 | 628.5384 | 2.38811 | 9.93181 | 0.04335 | 0.10069 | 4.39485E-4 | 6.8842 | 0.03005 | 70.164 | 0.99152 |
| 1150 |  | 8.77027 | 0.31204 | 693.0466 | 2.5166 | 9.85937 | 0.04086 | 0.10143 | 4.20353E-4 | 6.83399 | 0.02832 | 76.38346 | 0.99232 |
| 1200 |  | 8.30521 | 0.26871 | 464.8227 | 2.14375 | 9.90937 | 0.0524 | 0.10091 | 5.33595E-4 | 6.86865 | 0.03632 | 56.19394 | 0.98759 |
| 1250 |  | 7.87753 | 0.32197 | 650.9583 | 2.56102 | 9.92318 | 0.04481 | 0.10077 | 4.55113E-4 | 6.87822 | 0.03106 | 80.50169 | 0.99093 |
| 1300 |  | 7.8303 | 0.28244 | 558.44922 | 2.23211 | 9.95314 | 0.04579 | 0.10047 | 4.62227E-4 | 6.89899 | 0.03174 | 61.65375 | 0.9906 |
| 1350 |  | 7.7116 | 0.23211 | 337.47421 | 1.89054 | 9.81387 | 0.06249 | 0.1019 | 6.48832E-4 | 6.80246 | 0.04332 | 42.56683 | 0.98201 |

**Table S1** Parameters of the monoexponential fits applied to emission decay traces of the emission of **1^Bi^** in *n*-hexane at r.t. at c = 22 µM.


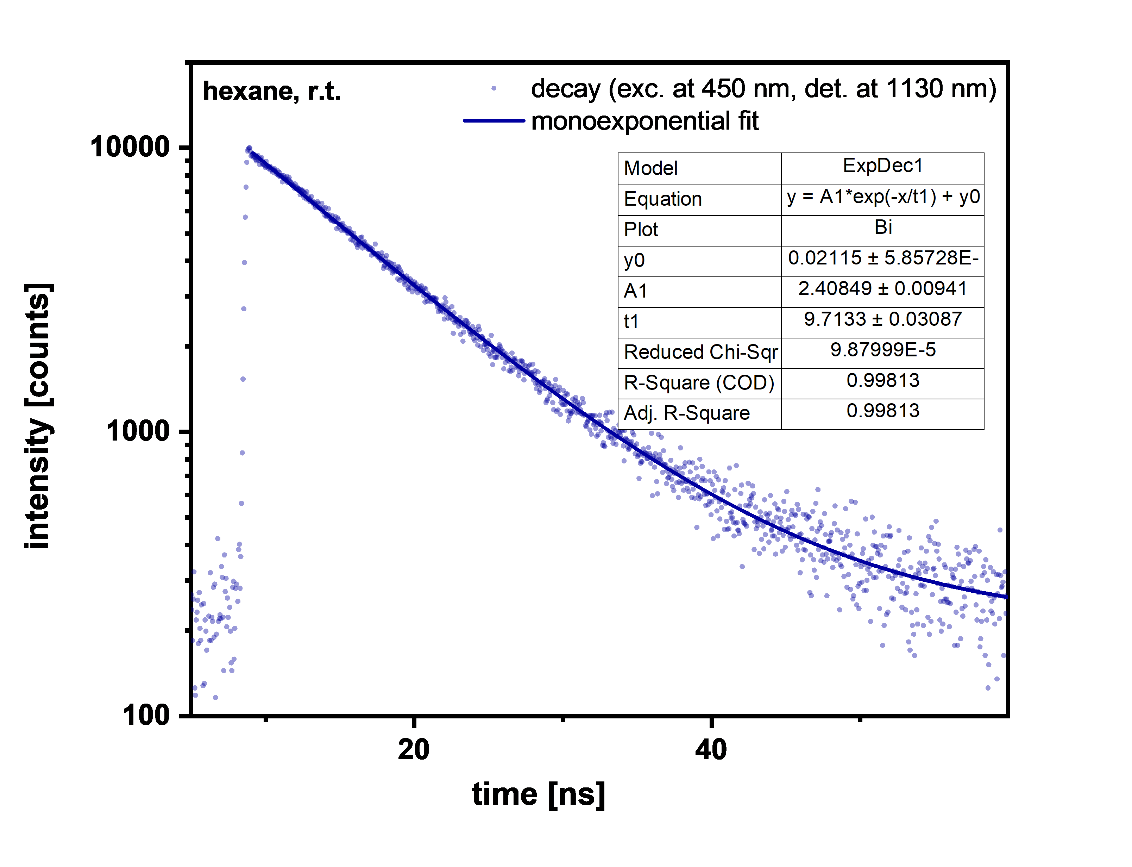


**Figure S21.** Emission decay trace of the phosphorescence of **1^Bi^**, hexane, r.t., ca. 22 µM


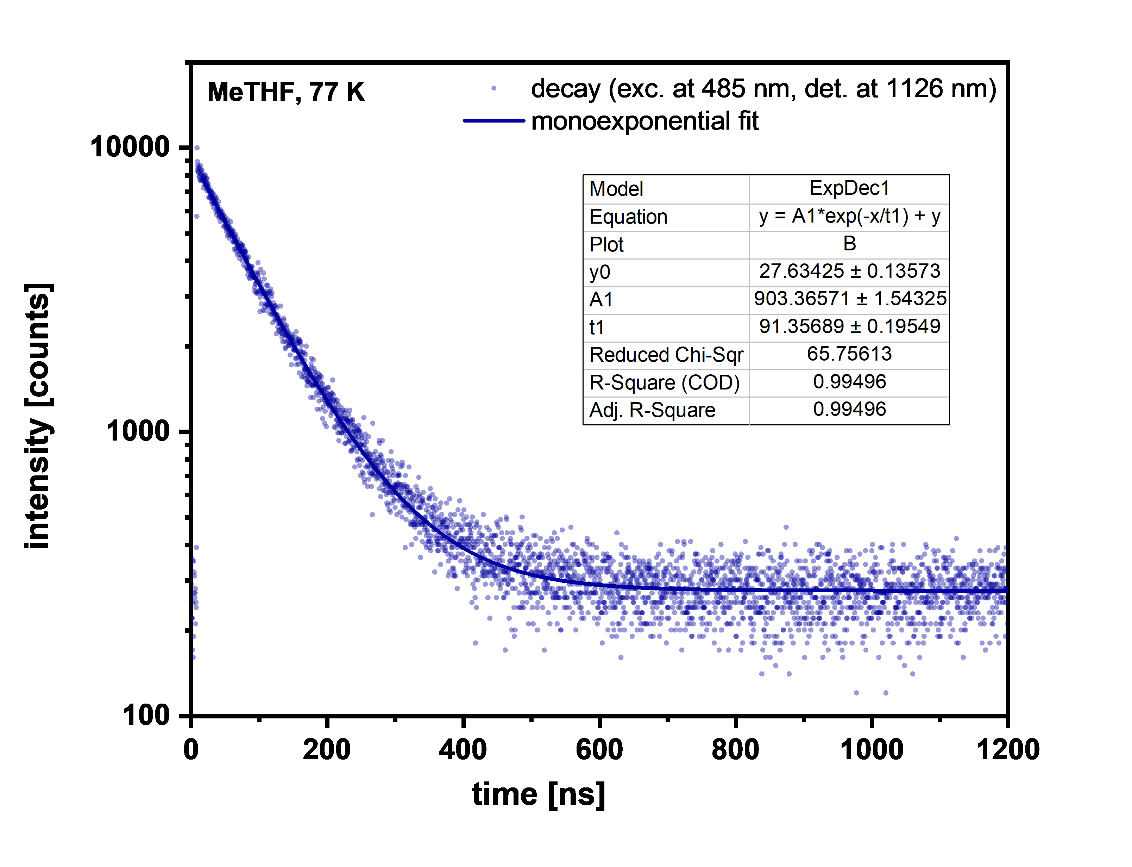


**Figure S22.** Emission decay trace of the phosphorescence of **1^Bi^**, MeTHF, 77K, ca. 22 µM

Figure S23. UV-Vis absorption spectra of solutions of 1^Sb^ recorded in *n*-hexane at different concentrations at r.t.

Figure S24. UV-Vis absorption spectra of solutions of 1^Sb^ recorded in different solvents at r.t.


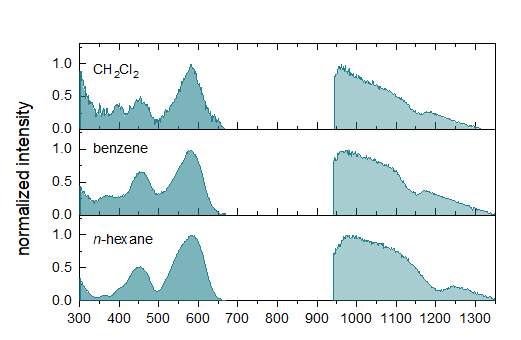


Figure S25. Emission (excited at 485 nm) and excitation spectra (detected at 1000 nm) of 1^Sb^ in differing solvents at r.t. at ca. *c* = 40 µM.

Figure S26. Emission and excitation spectra of 1^Sb^ in *n*-hexane at r.t. at differing concentrations.


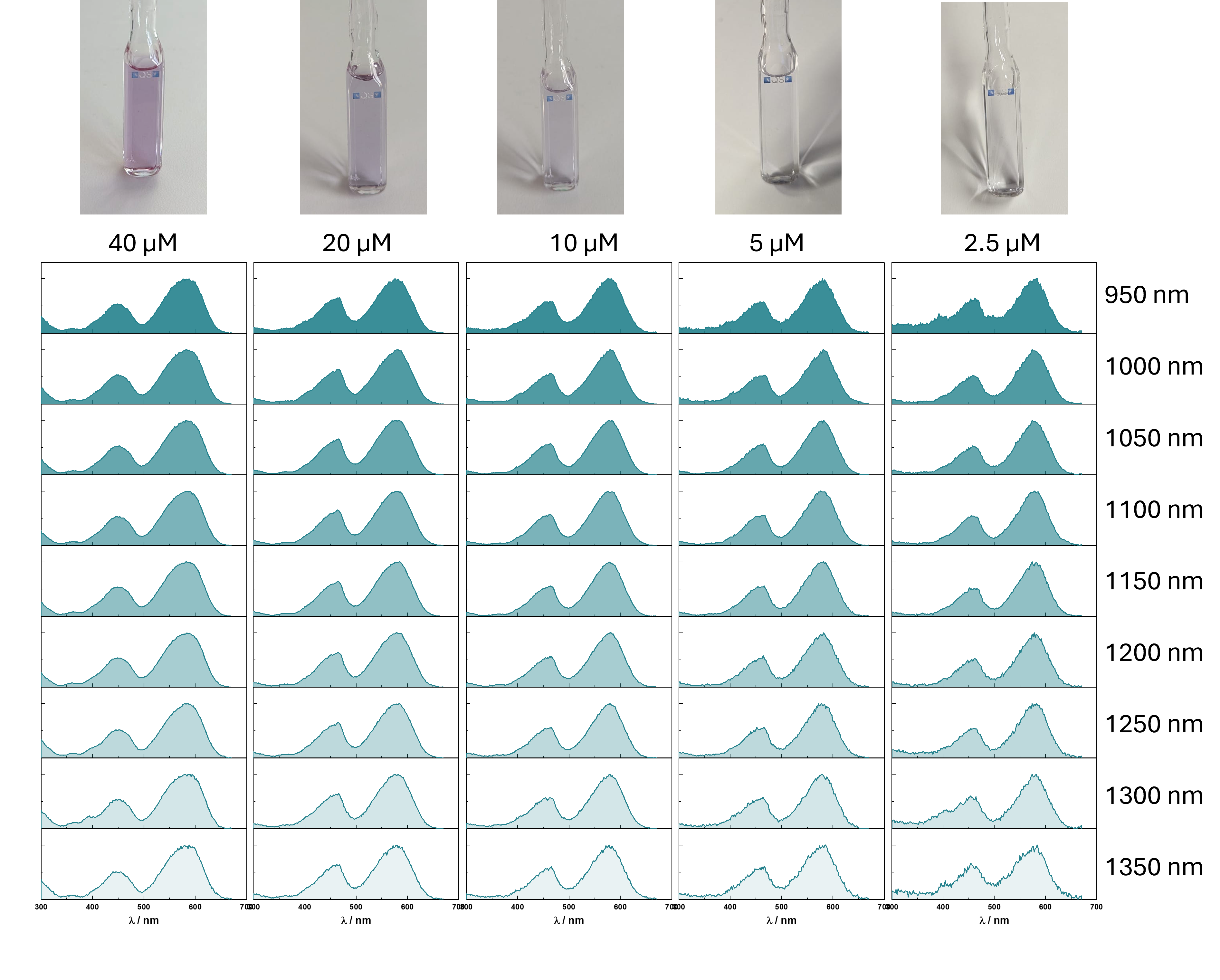


Figure S27. Normalized excitation spectra of the emission of 1^Sb^ detected at different wavelengths and concentrations in *n*-hexane at r.t.

Figure S28. Excitation spectra of the emission of 1^Sb^ recorded at different wavelengths in *n*‑hexane at r.t. c = 40 µM.

Figure S29. Left: Emission decay traces of the emission of 1^Sb^ recorded at different wavelengths in *n*‑hexane at r.t. c = 40 µM. Right: Intensity of the emission decay traces at *t* = 10 ns, and lifetimes extracted from emission decay traces at differing detection wavelengths.

Figure S30. Emission decay traces and monoexponential fits of the emission of 1^Sb^ recorded at different wavelengths in *n*‑hexane at r.t. c = 40 µM.

| **λ_det_** |  | **y0** | | **A1** | | **t1** | | | **k** | | | | **tau** | | | | **Statistics** | | | |
| --- | --- | --- | --- | --- | --- | --- | --- | --- | --- | --- | --- | --- | --- | --- | --- | --- | --- | --- | --- | --- |
|  |  | Value | Standard Error | Value | Standard Error | Value | Standard Error | | Value | Standard Error | | Value | | Standard Error | | Reduced Chi-Sqr | | Adj. R-Square | |  |
| 950 |  | 43.00719 | 0.26494 | 61.34077 | 1.49282 | 1.0381E-7 | | 3.80525E-9 | 9633021.1356 | | 353108.48211 | | 7.19553E-8 | | 2.6376E-9 | | 50.51988 | | 0.72102 | |
| 1000 |  | 42.50661 | 0.30943 | 128.19446 | 1.60242 | 1.12504E-7 | | 2.17424E-9 | 8888608.93179 | | 171780.64326 | | 7.79815E-8 | | 1.50707E-9 | | 64.83417 | | 0.90434 | |
| 1050 |  | 41.75457 | 0.30593 | 171.47113 | 1.55917 | 1.14195E-7 | | 1.61362E-9 | 8756988.22835 | | 123740.02368 | | 7.91536E-8 | | 1.11847E-9 | | 62.60642 | | 0.94665 | |
| 1100 |  | 42.72945 | 0.29482 | 177.20215 | 1.49523 | 1.14714E-7 | | 1.50657E-9 | 8717345.92272 | | 114487.81587 | | 7.95136E-8 | | 1.04428E-9 | | 57.92312 | | 0.95363 | |
| 1150 |  | 42.54327 | 0.30671 | 141.93682 | 1.5223 | 1.17023E-7 | | 1.96724E-9 | 8545352.23396 | | 143654.00769 | | 8.11139E-8 | | 1.36359E-9 | | 61.63751 | | 0.92653 | |
| 1200 |  | 42.72277 | 0.26846 | 79.92064 | 1.39062 | 1.12474E-7 | | 3.02551E-9 | 8890912.76366 | | 239161.59855 | | 7.79613E-8 | | 2.09712E-9 | | 48.81124 | | 0.8299 | |
| 1250 |  | 43.04359 | 0.28776 | 94.20874 | 1.47824 | 1.13354E-7 | | 2.75702E-9 | 8821945.90653 | | 214569.55118 | | 7.85708E-8 | | 1.91102E-9 | | 55.72855 | | 0.8567 | |
| 1300 |  | 43.81078 | 0.28301 | 68.75517 | 1.4057 | 1.16944E-7 | | 3.74664E-9 | 8551121.51043 | | 273960.57541 | | 8.10592E-8 | | 2.59697E-9 | | 52.51016 | | 0.77631 | |
| 1350 |  | 43.57026 | 0.27111 | 36.92611 | 1.32886 | 1.18372E-7 | | 6.7044E-9 | 8447947.74377 | | 478478.68219 | | 8.20492E-8 | | 4.64714E-9 | | 47.67994 | | 0.52667 | |

**Table S1** Parameters of the monoexponential fits applied to emission decay traces of the emission of **1^Sb^** in *n*-hexane at r.t. at c = 40 µM.

**
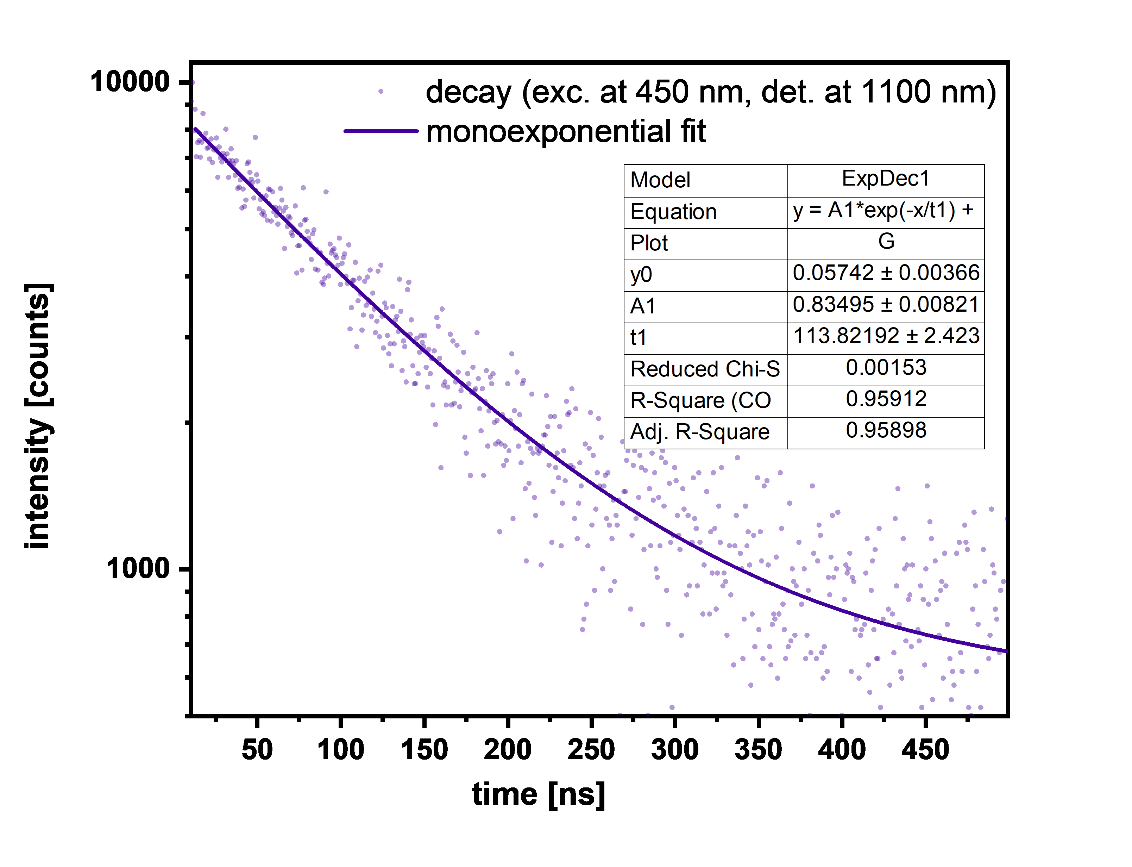
**

**Figure S31.** Emission decay trace of the phosphorescence of **1^Sb^**, hexane, r.t., 40 µM

**
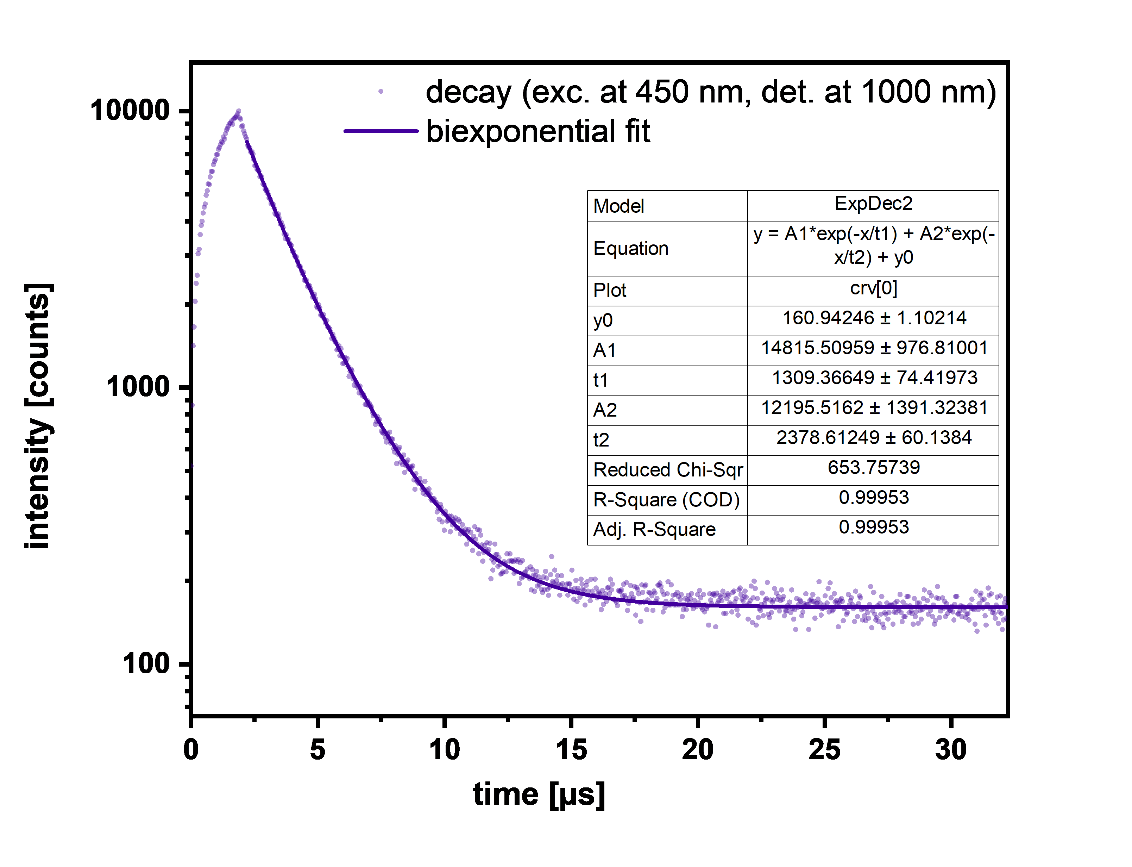
**

**Figure S32.** Emission decay trace of the phosphorescence of **1^Sb^**, MeTHF, 77K, 40 µM


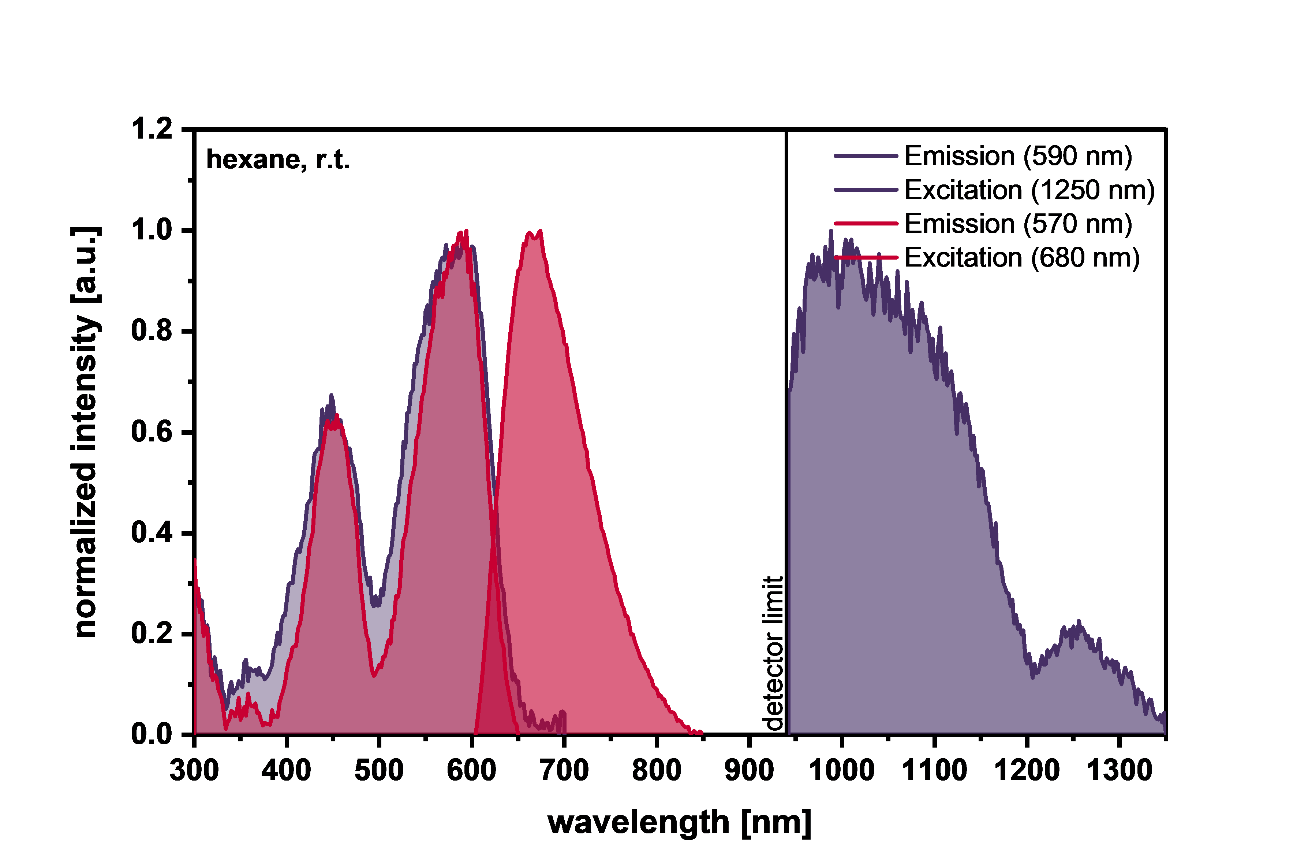


**Figure S33.** Fluorescence of **1^Sb^**, hexane, r.t, 40 µM.


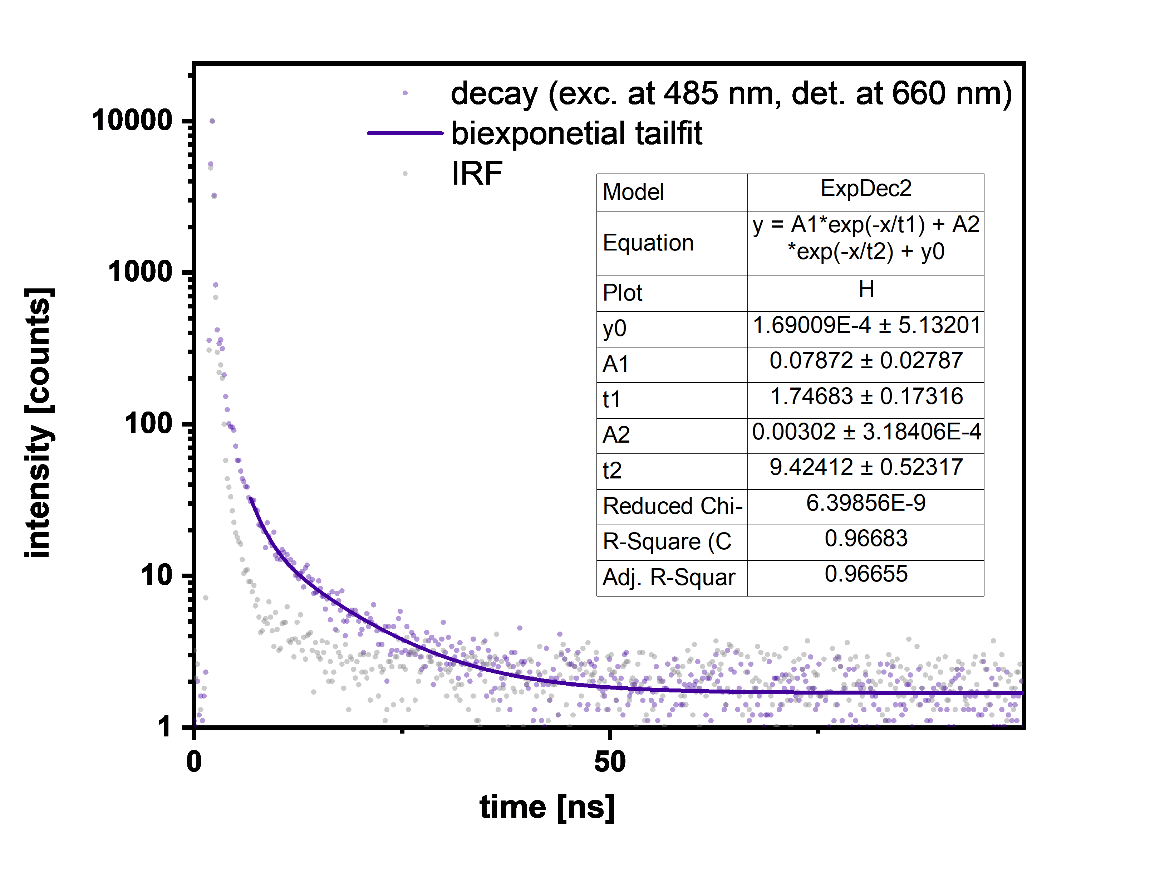


**Figure S34.** Lifetime of the fluorescence of **1^Sb^**, hexane, r.t., 40 µM.


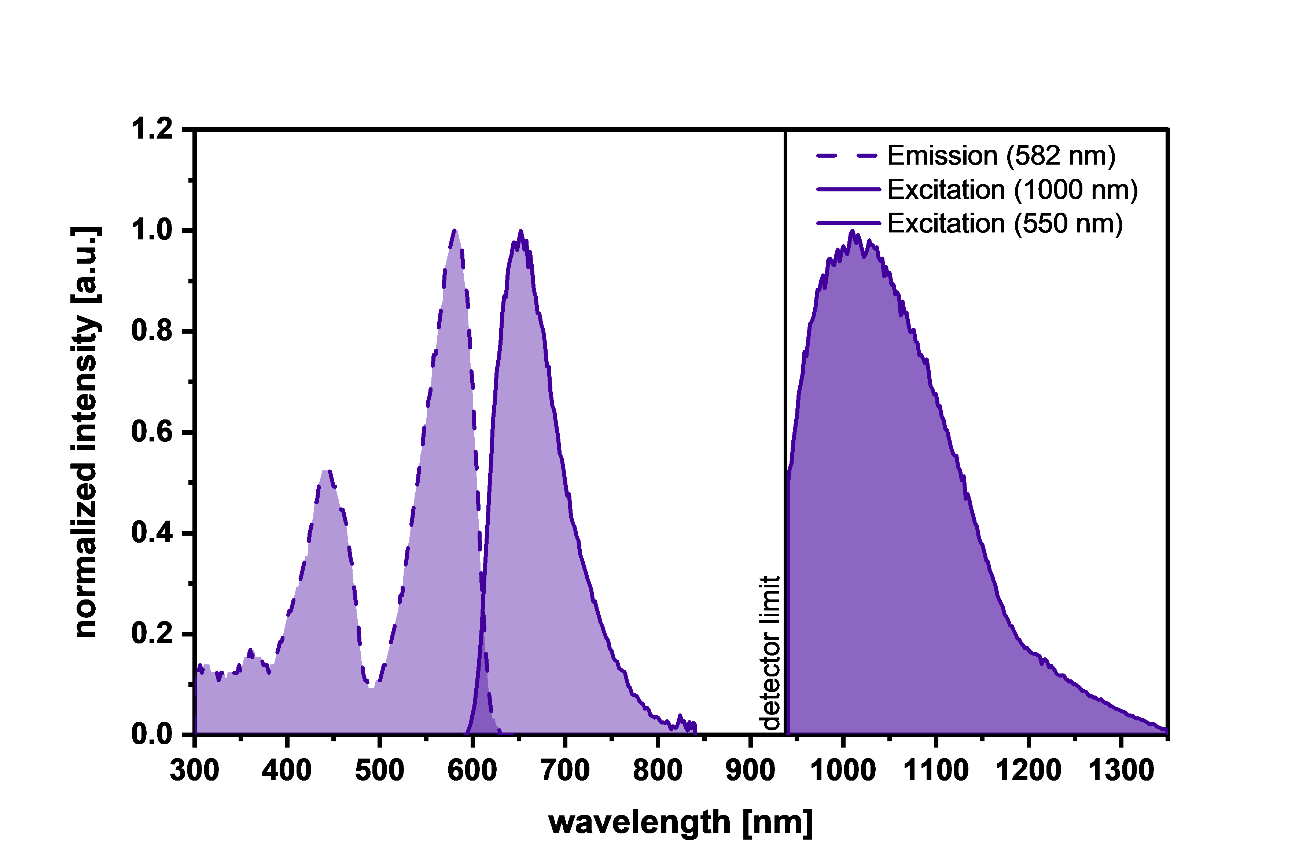


**Figure S35.** Fluorescence (left) and phosphorescence (right) of **1^Sb^**, MeTHF, 77K, 40 µM.

Figure S36. UV-Vis absorption spectra of 1^Bi^ and 1^Sb^ in *n*-hexane at r.t.

# Density functional theory calculations

1. **General Methods**

All calculations were carried out with the ORCA program package.^[11]^ All calculations were carried out on isolated molecules (in the gas phase). For computational efficiency, all methyl groups were replaced by hydrogen atoms. The RIJCOSX^[12]^ approximation was used for all DFT, TDDFT and CASSCF calculations. All DFT calculations, including geometry optimisations, frequency analyses, TDDFT and excited state dynamics, were performed using wB97X-D3^[13]^ ZORA-def2-TZVP^[14]^ (SARC-ZORA-TZVP on Sb^[15]^ and Bi^[16]^) together with the ZORA Hamiltonian to account for relativistic effects. The same basis set combination was used for the CASSCF calculations. For spin-orbit coupling calculations, the RI-SOMF(1X)^[17]^ operator was used.


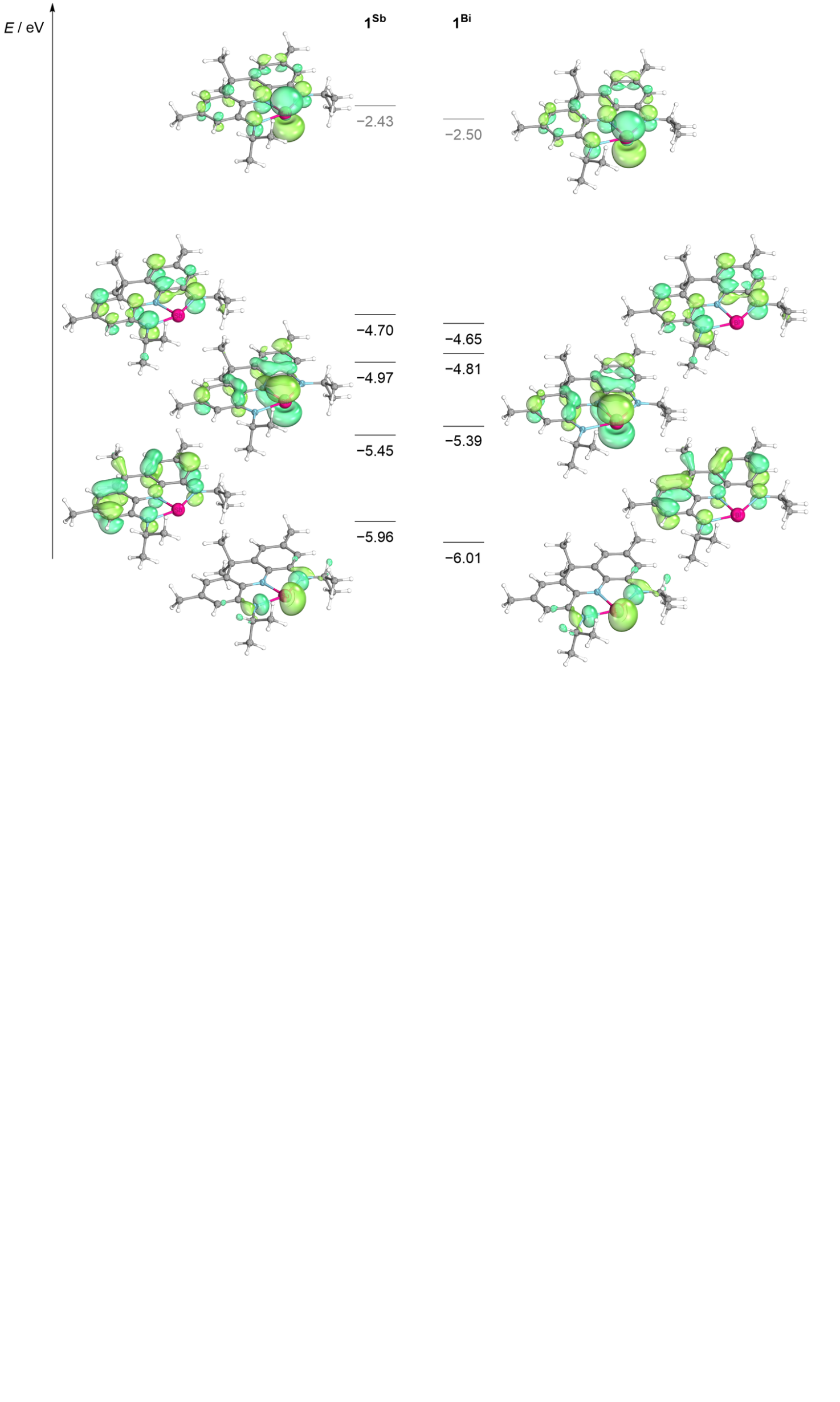


Figure S37. Frontier molecular orbitals of 1^Sb^ and 1^Bi^.

1. **CASSCF calculations**

As shown in earlier works on a related bismuth system, an active space of two electrons in two orbitals is suitable to describe the bonding between the pnictogen atoms and the central nitrogen center (CAS(2,2)).^[18]^ The initial orbitals were derived from DFT calculations and represent the Pn-N π-bonding and π-antibonding orbitals. To determine the bond polarity, we transformed the active orbitals into localized orbitals, resulting in a nitrogen-centered p-type orbital and a pnictogen centered p-type orbital (Figure S38). The resulting wavefunction consists of three resonance structures, with the second structure (**II**) having a higher weight than the third structure (**III**), suggesting a polarization of the Pn–N bond towards nitrogen. However, the dominant resonance structure (**I**) indicates a non-polarized, covalent 2c-2e π-bond between Pn and N. The high covalency makes it challenging to assign a formal oxidation state to Pn, but the presence of a Pn(III) center is justified based on the higher weight of **II** compared to **III**.

| 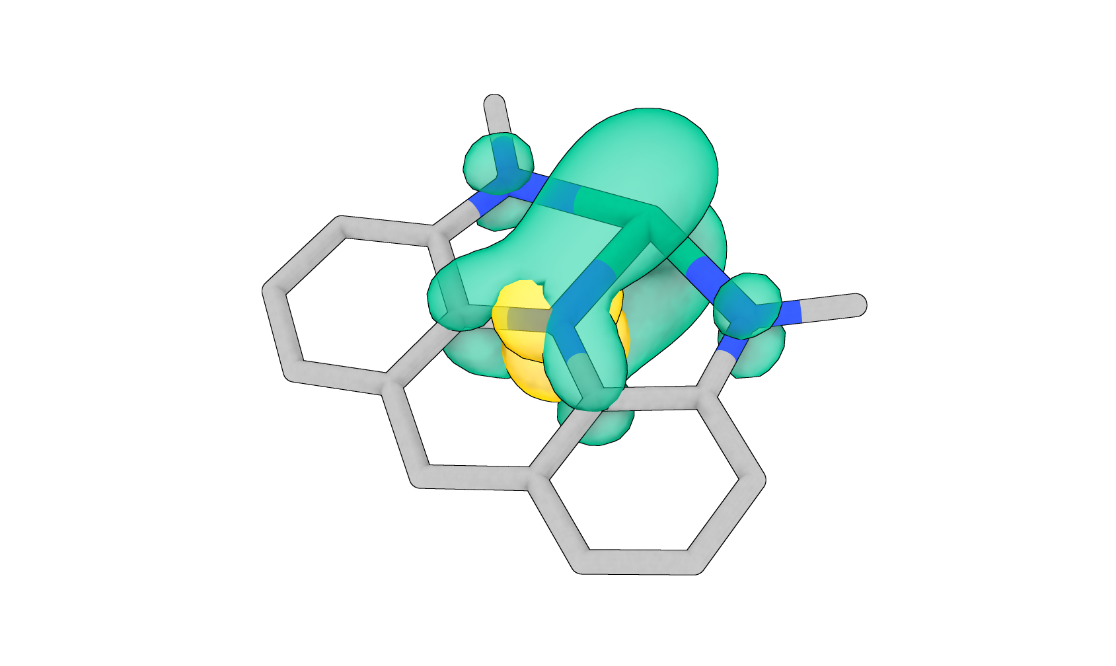 | 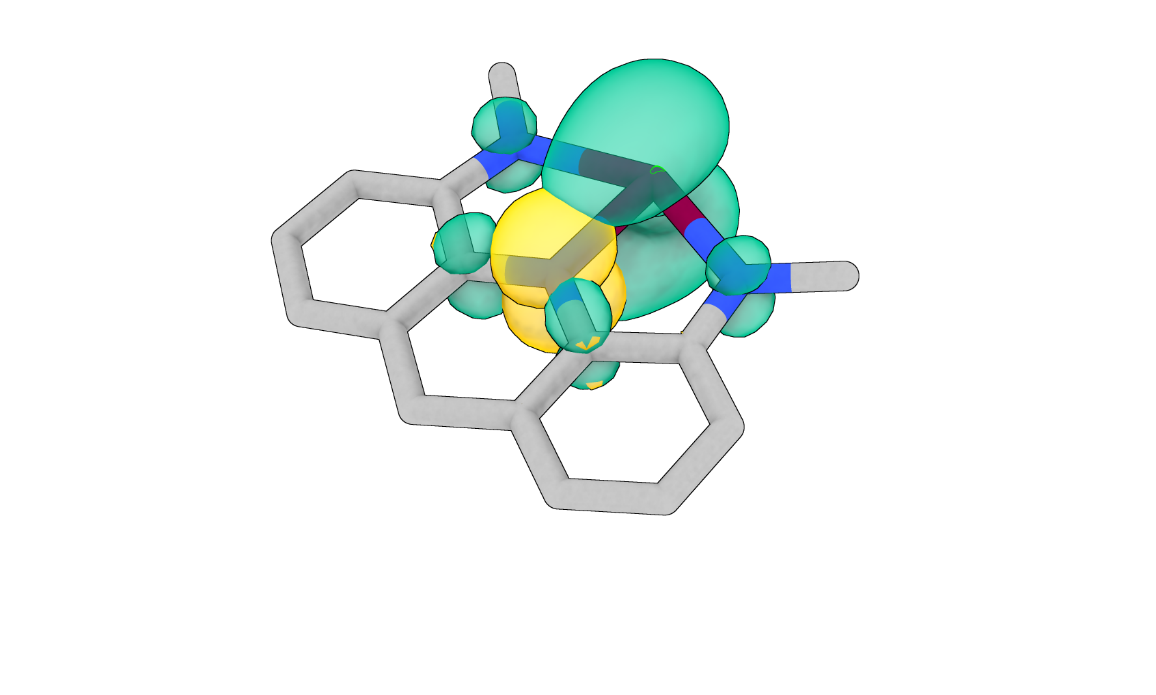 |
| --- | --- |
| **1^Sb^** | **1^Bi^** |

**Figure S38.** Localized CASSCF orbitals for **1^Sb^** and **1^Bi^**. Surface isovalue = 0.05. For clarity, both orbitals of the CAS space are shown, the nitrogen centered orbital is colored in yellow, the pnictogen-centered one is colored in green.

Figure S39. Resonance structures and their relative weights derived from CAS(2,2) calculations with localized orbitals.

1. **TDDFT calculations**

TDDFT calculations were performed including four excited singlet and triplet states, respectively, and the results are summarized in Table S5. The calculated spin–orbit coupling matrix elements (SOCME) obtained from the ORCA output are shown in Figures S40 and S41. As expected, larger SOCME values are observed for Pn = Bi due to its higher atomic mass compared to Sb. However, in the case of Pn = Sb, the near-degeneracy of the S_2_ and T_4_ states gives rise to a strong mixing of the two states. This results in two spin–orbit coupled states which exhibit significant oscillator strengths, ultimately manifesting as a characteristic double-band structure. This double-band structure is also visible in the gaussian deconvolution of the experimental spectra. The line-fitting of Sb requires 8 gaussian functions to fit the two intense absorptions in the visible region, whereas Bi only requires 6 functions (Figure S42 and S43).

**Table S1** Energies of excited states relative to the ground state S_0_ in cm^–1^.

|  | **S_1_** | **S_2_** | **S_3_** | **S_4_** | **T_1_** | **T_2_** | **T_3_** | **T_4_** |
| --- | --- | --- | --- | --- | --- | --- | --- | --- |
| **1^Sb^** | 22219 | 25657 | 30173 | 30551 | 13621 | 14526 | 22911 | 25300 |
| **1^Bi^** | 20259 | 23116 | 28586 | 307967 | 10566 | 12226 | 21360 | 28002 |


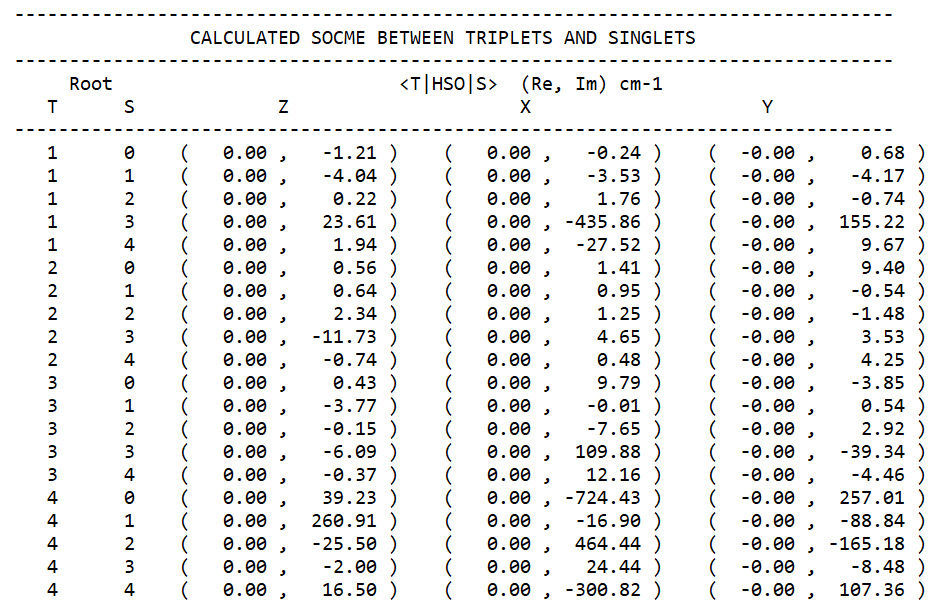


Figure S40. Calculated SOCME for 1^Sb^.


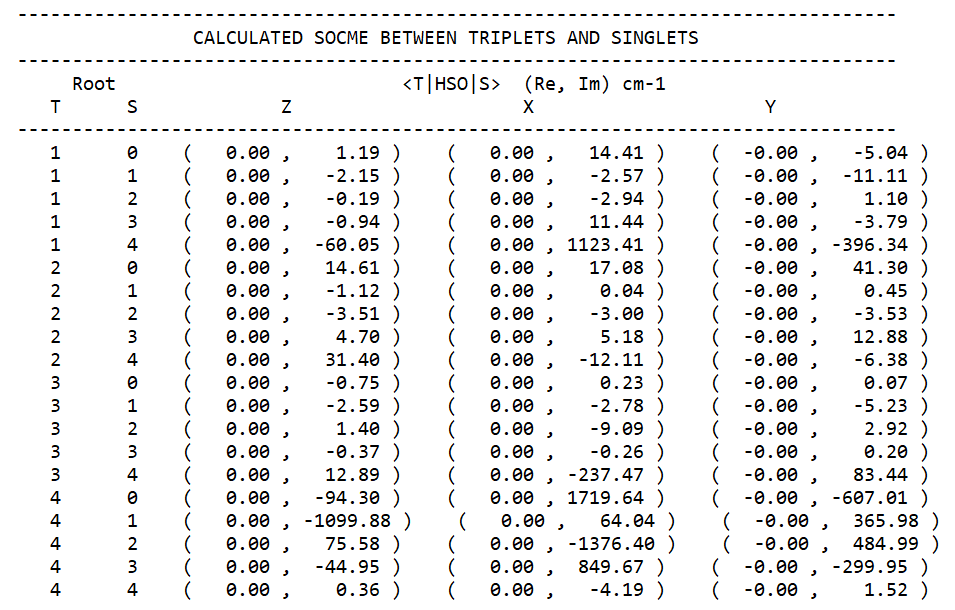
 Figure S41. Calculated SOCME for 1^Bi^.


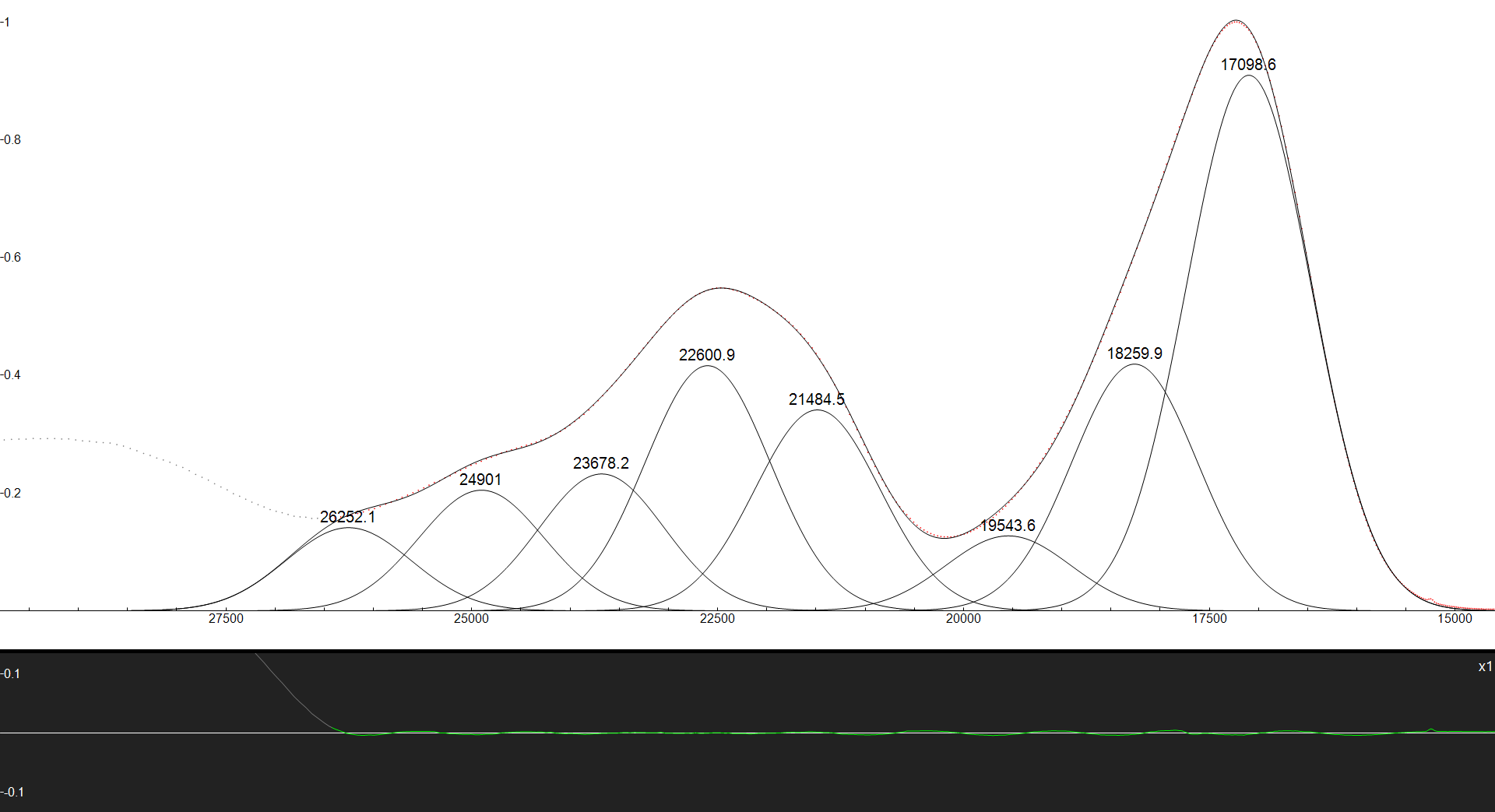


Figure S42. Section of the UV-Vis spectrum of 1^Sb^ (dotted red line). The fit functions are shown as black curves together with their center. FWHM = 1500 cm^–1^.


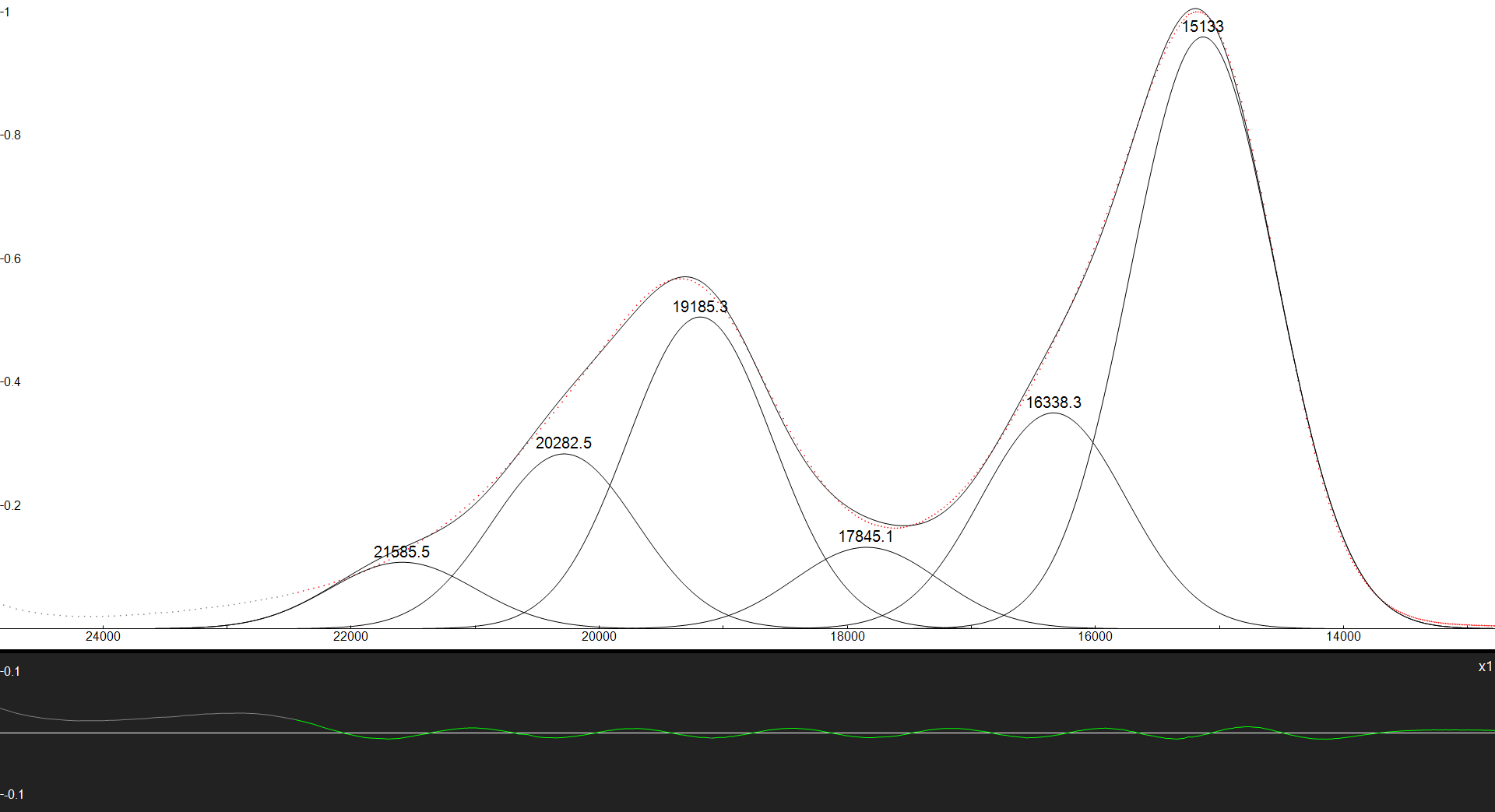


Figure S43. Section of the UV-Vis spectrum of 1^Bi^ (dotted red line). The fit functions are shown as black curves together with their center. FWHM = 1350 cm^–1^.

1. **Calculation of phosphorescence spectra**

Phosphorescence spectra were calculated using the excited state dynamics module of ORCA. Specifically, the structures and Hessians of the S_0_ and T_1_ states were used as input and Herzber-Teller contributions and Duchinsky rotations were considered. The resulting calculated spectra are shown below. Here, the maximum wavelengths of the emissions are in good agreement with the experiment (**1^Sb^**: 1085 nm, **1^Bi^**: 1270 nm). However, the double-band structure is not reproduced.

Figure S44. Calculated phosphorescence spectra of 1^Sb^ (blue) and 1^Bi^ (green), intensities are normalized.

# References

[1] S. Pavlidis, J. Alasadi, A. Opis-Basilio, J. Abbenseth, *Dalton Trans.* **2025**, *54*, 2421–2429.

[2] C. J. Carmalt, N. A. Compton, R. J. Errington, G. A. Fisher, I. Moenandar, N. C. Norman, K. H. Whitmire in *Inorg. Synth.***1996**, *31*, 98 – 101.

[3] H. Schumann, *J. Organomet. Chem.* **1986**, *299*, 169–178.

[4] S. Pavlidis, E. W. Fischer, A. Opis-Basilio, A. Bera, A. G. Buzanich, S. Wittek, F. Emmerling, K. Ray, M. Roemelt, J. Abbenseth, *J. Am. Chem. Soc.* **2026** DOI: 10.1021/jacs.5c18955.

[5] SAINT. Bruker AXS Inc., Madison, Wisconsin, USA.

[6] L. Krause, R. Herbst-Irmer, G. M. Sheldrick, D. Stalke *J. Appl. Crystallogr.* **2015**, *48*, 3–10.

[7] G. M. Sheldrick *Acta Crystallogr. Sect. Found. Adv.* **2015**, *71*, 3–8.

[8] G. M. Sheldrick *Acta Crystallogr. Sect. C Struct. Chem.* **2015**, *71*, 3–8.

[9] C. R. Groom, I. J. Bruno, M. P. Lightfoot, S. C. Ward *Acta Crystallogr. Sect. B Struct. Sci. Cryst. Eng. Mater.* **2016**, *72*, 171–179.

[10] D. Kratzert *Finalcif_V152* [**https://dkratzert.de/finalcif.html**](https://dkratzert.de/finalcif.html).

[11] F. Neese, F. Wennmohs, U. Becker, C. Riplinger, *J. Chem. Phys.* **2020**, *152*, 224108.

[12] F. Neese, F. Wennmohs, A. Hansen, U. Becker, *Chem. Phys.* **2009**, *356*, 98–109.

[13] Y.-S. Lin, G.-D. Li, S.-P. Mao, J.-D. Chai, *J. Chem. Theory Comput.* **2013**, *9*, 263–272.

[14] F. Weigend, R. Ahlrichs, *Phys. Chem. Chem. Phys.* **2005**, *7*, 3297–3305.

[15] J. D. Rolfes, F. Neese, D. A. Pantazis, *J. Comput. Chem.* **2020**, *41*, 1842–1849.

[16] D. A. Pantazis, F. Neese, *Theor. Chem. Acc.* **2012**, *131*, 1292.

[17] F. Neese, *J. Chem. Phys.* **2005**, *122*, 034107.

[18] P. Coburger, A. Guilherme Buzanich, F. Emmerling, J. Abbenseth, *Chem. Sci.* **2024**, *15*, 6036–6043.
